# Nonequilibrium Navigation of a Rugged Energy Landscape Enables the Formation of Face-On Single-Domain Dodecagonal Quasicrystals

**DOI:** 10.1021/jacs.6c09814

**Published:** 2026-09-30

**Authors:** Mayumi Egashira, Jing-Wei Chen, Mao-Yan Wang, Chun-Jen Su, Yi-Qi Yeh, Wei-Tsung Chuang, Chien-Lung Wang

**Affiliations:** † Department of Chemistry, 202948 National Taiwan University, No. 1, Sec. 4, Roosevelt Rd, Taipei 10617, Taiwan; ‡ 57815 National Synchrotron Radiation Research Center, 101 Hsin-Ann Road, Hsinchu 30076, Taiwan

## Abstract

Metastable intermediates play an important role in directing structural evolution across complex energy landscapes. Here, we establish the dodecagonal quasicrystal (DDQC) as a tunable metastable kinetic junction in a single-component carboxyl-functionalized dendritic assembly. Time-resolved small-angle X-ray scattering (SAXS) reveals that this DDQC junction bifurcates into distinct Frank–Kasper (FK) phases through competing kinetic pathways: a deformation-dominated route to the A15 phase at lower temperatures or a mass-exchange-mediated transformation to the thermodynamically favored σ phase at higher temperatures. By introducing amine-mediated salt bridges to rigidify the micellar cores, we selectively suppress the deformation-driven A15 pathway while preserving access to the σ phase, thereby programming the bifurcation behavior of the DDQC junction. Leveraging this core engineering, a vapor-induced self-assembly (VISA) strategy was further developed to enable a nonequilibrium σ-to-DDQC transition. The high nucleation barrier inherent to this nonequilibrium pathway creates a nucleation-controlled regime that suppresses spontaneous polydomain formation, enabling the growth of macroscopically aligned, face-on single-domain DDQC with persistent 12-fold symmetry over millimeter length scales. These findings demonstrate that micellar core engineering provides a powerful platform for navigating rugged energy landscapes, transforming metastability from an uncontrolled kinetic trap into a programmable element for deterministic pathway selection and long-range quasiperiodic order in soft matter.

## Graphical abstract

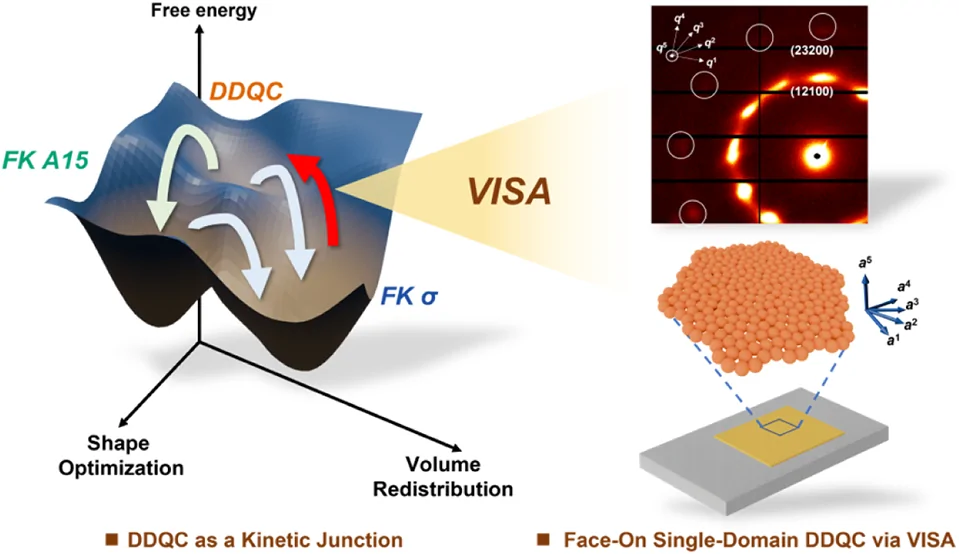

## Introduction

Metastable intermediates are central to how complex systems organize and evolve.^1^ In biological contexts such as protein folding, the assembly process is governed by a rugged energy landscape, where the molecule must navigate through a multitude of local minima and conformational states to reach its native structure. This intricate balance between enthalpic stabilization and entropic frustration allows for a rich structural diversity and pathway complexity.^2,3^ This principle extends beyond biology and finds an analogue in self-assembled soft materials.^4–6^

In many soft condensed matter systems, self-organization can be understood in terms of the competition between maximizing entropy and minimizing interfacial area.^7,8^ This competition often gives rise to complex energy landscapes, leading soft matter systems to evolve through long-lived, ordered metastable configurations that precede their equilibrium phases. Among these, the dodecagonal quasicrystal (DDQC) has emerged as an emblematic example. Exhibiting crystallographically forbidden 12-fold rotational symmetry without periodic translational order, the DDQC represents a unique form of order distinct from both periodic crystals and amorphous materials.^9^ First discovered in dendritic liquid crystals (LCs),^10^ the DDQC phase was subsequently observed in a wide range of soft matter systems, including block copolymers,^11–14^ giant molecules,^15–17^ glycolipids,^18,19^ surfactants,^20^ T-shaped polyphiles,^21,22^ colloidal systems,^23–26^ and DNA nanostructures.^27^

Theoretical and experimental studies have established close structural relationships between the DDQC and Frank–Kasper (FK) phases—a family of tetrahedrally close-packed (t.c.p.) lattices constructed from polyhedral coordination environments.^28,29^ In particular, the A15 (*Pm* 3̅ *n*) and σ (*P4*_2_ */mnm*) structures share local geometries that can be derived from the same square–triangle tiling framework underlying the DDQC.^10,30^ In many soft-matter systems, the DDQC has been experimentally observed as a kinetically favored precursor to the σ phase.^12,15,31^

While the structural kinship between DDQC and FK phases is well documented, deterministically controlling the selection between competing FK minima remains a challenge. Theoretical calculations indicate that the free-energy difference between the A15 and σ phases is small (Δ*F* ≈ 10^–4^ *k_B_T* per chain) such that even subtle variations in interfacial energy or chain stretching can shift stability among competing states.^32,33^ Consequently, access to A15, σ, and DDQC phases in soft-matter assemblies has so far relied largely on composition control, where changes in molecular architecture or component ratios guide the system toward different free-energy minima,^34–38^ and direct observation of all three phases within both single-component^39,40^ and multicomponent systems^19^ remains rare. Moreover, previous studies primarily relied on observing structural evolution under continuously varying temperatures, a protocol that can conflate changes in thermodynamic stability with kinetic processes, making it difficult to isolate these structures as pure phases.

Beyond serving as a metastable intermediate en route to periodic FK phases, the quasiperiodic plane of the DDQC, with its intrinsic 12-fold rotational symmetry, provides a unique platform for isotropic photonic bandgaps and unconventional wave phenomena that cannot be attained in periodic crystals.^41^ However, exposing this quasiperiodic plane over large, macroscopically coherent domains remains a challenge in soft-matter systems due to inherent kinetic and thermodynamic constraints. As a kinetically favored intermediate, the DDQC is prone to rapid nucleation, often leading to the formation of randomly oriented domains.^42^ In bulk soft-matter systems, the DDQC inherently favors an edge-on alignment, predominantly exposing its out-of-plane periodic stacking and restricting direct access to the quasiperiodic plane.^10,43^ As a result, precise control over both macroscopic face-on alignment and nucleation kinetics in DDQC supramolecular assemblies remains to be highly challenging. To overcome these limitations, pioneering efforts have been explored for various alignment strategies. While specific surface treatments can induce face-on orientations in thin films, the observation of quasiperiodic order has primarily been confined to localized microscopic regions, leaving large-domain alignment as an ongoing challenge.^43^ Likewise, macroscopic external stimuli, such as shear fields, often yield complex, off-axis multidomain structures with limited structural coherence.^14^ Under these conditions, the observed scattering or imaging signatures are heavily convoluted by out-of-plane periodic stacking, thereby obscuring intrinsic quasiperiodic tiling. Consequently, large-domain quasiperiodic order in DDQCs remains largely elusive on the macroscopic scale, posing a critical barrier to both structural elucidation and its exploitation in advanced macroscopic applications, such as photonic quasicrystals^41^ and optical metamaterials.^44^

To clarify the role of the intermediate DDQC within a rugged energy landscape toward FK phases and elucidate the kinetic pathway leading to face-on DDQC morphology, we propose that micellar core engineering enables programmable control over the underlying kinetic pathways, including micellar shape optimization via deformation^45–47^ and volume redistribution via intermicellar mass exchange.^48^ However, conventional rigid aromatic cores in dendritic self-assembly impose intrinsic geometric constraints that hinder these necessary rearrangements.^10,34,49^ To rationally access and regulate these distinct kinetic pathways, we employed a carboxyl-functionalized dendron (**CD**) based on a pentaerythritol core, as shown in Scheme 1a. Unlike rigid aromatic centers, this aliphatic core imparts conformational flexibility, providing the requisite deformability for shape optimization. Furthermore, the carboxylic core unit renders the strength of noncovalent interactions tunable via temperature, while the incorporation of amine-based guests establishes reversible salt bridges^50,51^ within the dendron cores to modulate the mechanical rigidity^52^ of the micelles. This tunability allows for the precise manipulation of the DDQC–FK energy landscape, thereby governing the metastability of the DDQC phase and regulating the competition between different kinetic routes. In this work, we utilize this programmable platform to investigate the pivotal role of the DDQC as a metastable kinetic junction. By employing time-resolved small-angle X-ray scattering (SAXS) under isothermal conditions, we elucidate the thermally accessible DDQC → A15 and DDQC → σ transformation pathways. Specifically, increasing micellar rigidity effectively suppresses the deformation-mediated transformation to the A15 phase (Scheme 1b). This precise control over amine uptake allows us to demonstrate a vapor-induced self-assembly (VISA) process that drives a nonequilibrium σ → DDQC phase transition. The elevated kinetic barrier associated with this nonequilibrium pathway suppresses excessive nucleation. By exploiting this kinetically restricted regime, the formation of macroscopically aligned, face-on single-domain DDQC is enabled. Collectively, these design principles establish the pivotal role of the DDQC as a metastable kinetic junction and provide a general strategy for harnessing metastability to achieve deterministic pathway selection, nucleation control, and macroscopic structural ordering in complex soft-matter systems.

**Scheme 1:**
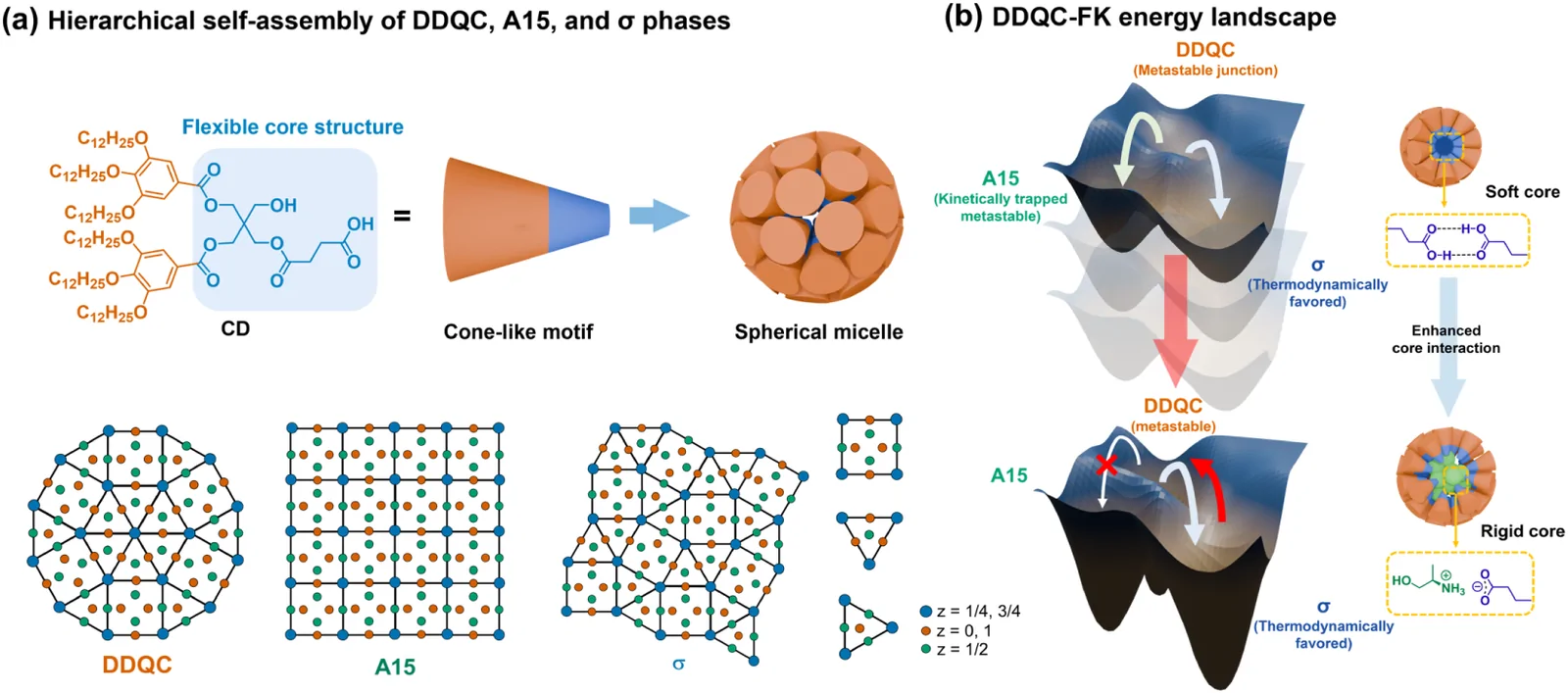
Hierarchical Self-Assembly and the Tunable Energy-Landscape Framework of the CD System^a^

a (a) The **CD** molecule adopts a cone-like motif to form spherical micelles, which further organize into DDQC and FK phases (A15 and σ). (b) Schematic of the DDQC–FK energy landscape, illustrating DDQC as a metastable kinetic junction connecting competing FK minima. Enhanced core interactions modulate the relative kinetic barriers and accessible transformation pathways with temperature. The nonequilibrium navigation driven by VISA demonstrates chemically regulated access to the reverse phase transition.

## Results and Discussion

### Thermotropic Phase Behavior of CD and Metastability of DDQC

The molecular design of **CD** consists of a pentaerythritol-based core as a central scaffold, functionalized with three alkyl-substituted benzoyl groups at the periphery. At the focal point, a carboxyl group is introduced to impart directional interactions, yielding a dendritic, cone-like architecture, as shown in Scheme 1a. Synthetic routes are detailed in Scheme S1, and the molecular structure was confirmed by ^1^H NMR, ^13^C NMR, and MS, as shown in Figures S1–S3.

The thermotropic phase behavior of **CD** was investigated by *in situ* temperature-dependent SAXS at a heating rate of 10 °C min^–1^ (Figure 1). At each temperature step, the sample was held for 3 min prior to measurement to allow thermal equilibration. These phase transitions were further corroborated by differential scanning calorimetry (DSC) (Figure S4). The initial DDQC phase was obtained from the as-prepared **CD** sample after complete solvent removal under vacuum. During the first heating scan (Figure 1a), the DDQC phase was observed from 30 to 50 °C. Upon further heating, the DDQC gradually transformed into the FK σ phase before isotropizing near 70 °C. In contrast, the second heating scan (Figure 1b) displayed only the σ phase across the entire temperature range. This monotropic phase behavior is consistent with the DDQC being metastable, existing as a kinetic intermediate during the initial heating process and irreversibly transforming into the thermodynamically favored σ phase. The diffraction peaks of the σ phase in the second heating scan appear broader compared to those in the first heating scan. The broader σ reflections in the second heating scan originate from partial structural disorder introduced during the first cooling process, as evidenced by the broadened σ peaks in Figure S5. The higher structural perfection of the σ phase in the first heating scan underscores the role of the DDQC as a pre-organized template that effectively guides the system across the complex energy landscape toward its highly ordered σ structure.

**Figure 1:**
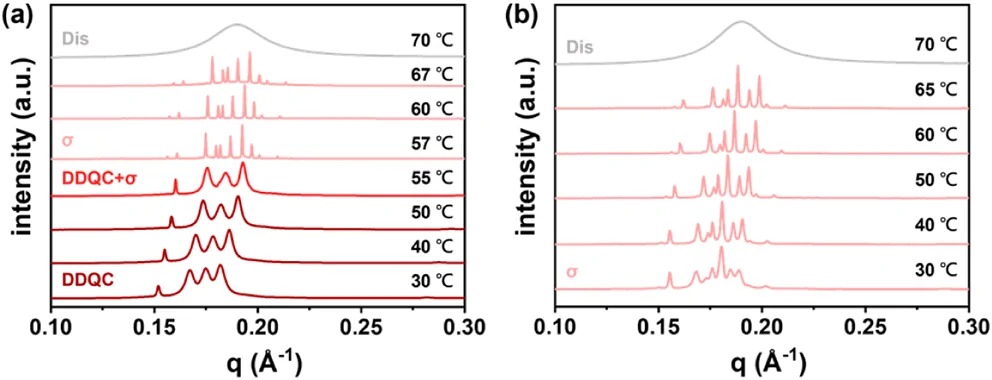
*In situ* temperature-dependent SAXS profiles of **CD** assemblies. (a) The first heating scan. (b) The second heating scan. The sample was heated stepwise (ramp rate: 10 °C min^–1^) and equilibrated for 3 min at each temperature prior to data acquisition.

### Isothermal Kinetic Pathways on the Competing Energy Landscape

The stability of ordered phases is governed by the Gibbs free energy, which is inherently temperature-dependent. Consequently, structural evolution observed under continuously varying temperature conflates changes in thermodynamic stability with kinetic processes, hindering a direct mapping of the energy landscape. To decouple these effects and rigorously probe the phase transition pathways, we monitored the structural evolution under isothermal conditions. The time-resolved SAXS profiles track the isothermal evolution of the **CD** assemblies at 40 and 50 °C (Figure 2). At 40 °C, the initially formed DDQC gradually transformed into a coexisting DDQC + A15 state within approximately 9 h, followed by a complete conversion to the A15 phase after 168 h (Figure 2a). In contrast, at 50 °C, the DDQC transformed into the σ phase through an intermediate DDQC + σ coexistence region within 3 h (Figure 2b). Representative SAXS patterns of the DDQC, A15, and σ phases are compiled in Figure 2c–e. These profiles are indexed to the DDQC, the cubic A15 (*Pm*3̅*n*), and the tetragonal σ (*P4*_*2*_ */mnm*) structures, respectively. A complete list of peak positions and assigned indices for each phase is provided in Tables S1–S3.

**Figure 2:**
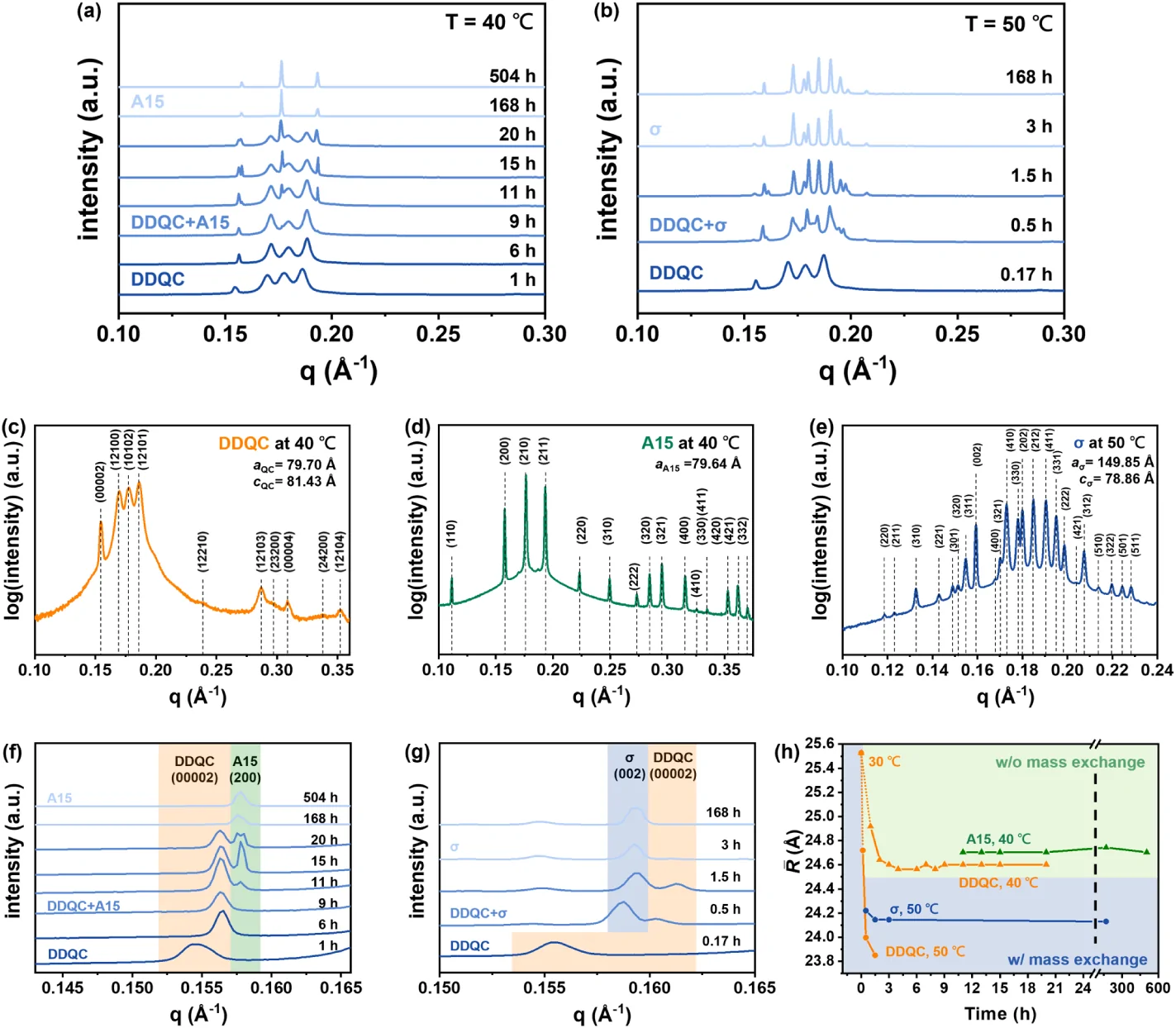
Time-resolved SAXS characterization of the isothermal evolution of **CD** assemblies. (a) SAXS patterns recorded during annealing at 40 °C. (b) SAXS patterns recorded during annealing at 50 °C. (c–e) Representative indexed SAXS profiles of the (c) DDQC phase at 40 °C, (d) A15 phase at 40 °C, and (e) σ phase at 50 °C. (f) Enlarged view of the DDQC (00002) and A15 (200) reflections during the DDQC → A15 transition at 40 °C. (g) Enlarged view of the DDQC (00002) and σ (002) reflections during the DDQC → σ transition at 50 °C. (h) Time evolution of the average particle radius (*R̅*) at 40 and 50 °C.

To highlight the structural signatures that distinguish these pathways, the key reflections associated with DDQC (00002), A15 (200), and σ (002) were magnified in Figure 2f,g. These peak positions serve as a metric for quantifying the evolution of the average particle radius (*R̅*), as shown in Figure 2h and Table S4. Detailed calculation procedures are provided in the Supporting Information (SI). Although the A15, σ, and DDQC phases contain multiple types of polyhedral micelles, the volume distributions within these specific structures remain relatively narrow. Previous studies on related giant-molecule assemblies have shown that A15 and σ are associated with a low-volume-asymmetry regime, with the largest-to-smallest mesoatom volume ratio (*V*_L_/*V*_S_) close to unity (<1.2 for A15 and approximately 1.2–1.35 for σ),^53^ while Wigner–Seitz-cell analysis has shown that DDQC exhibits a relatively narrow micellar-volume distribution that is nearly identical to that of σ.^16^ Accordingly, *R̅* provides a useful phase-averaged descriptor for tracking their structural evolution. The divergent trends in *R̅* during the DDQC → A15 and DDQC → σ transitions can be interpreted within a shared free-energy landscape, where the DDQC acts as a metastable kinetic junction (schematically illustrated in Figure 3). Raman spectroscopy, based on the analysis of characteristic *gauche* (*ν*(C–C)_G_ ≈ 1077 cm^–1^) and *trans* (*ν*(C–C)_T_ ≈ 1064 cm^–1^) bands,^54^ reveals a hierarchy of chain stretching: DDQC > σ > A15, indicating that the metastability of the DDQC phase stems from unfavorable chain stretching (Figure S6). The two pathways correspond to distinct modes of relieving interfacial and stretching penalties under different kinetic constraints.

**Figure 3:**
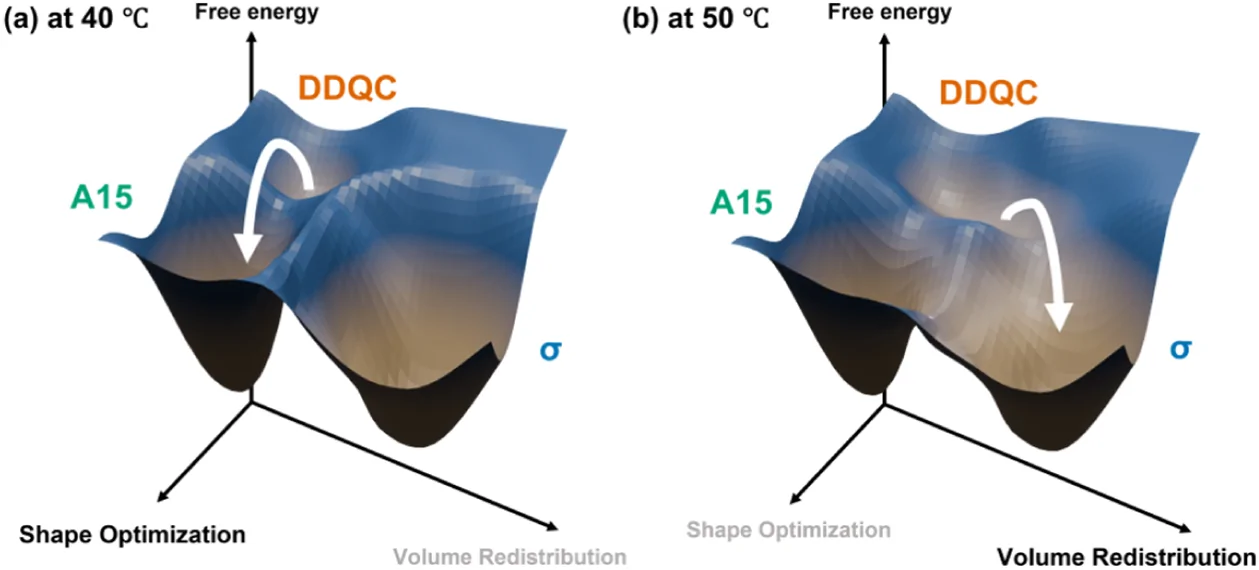
Schematic illustration of the temperature-dependent bifurcation in the free-energy landscape. (a) At 40 °C, the system evolves into the A15 phase via the shape optimization pathway. (b) At 50 °C, the system relaxes into the σ phase via the volume redistribution pathway.

During the DDQC → A15 transition at 40 °C, *R̅* remains nearly invariant (Δ*R̅* ≈ 0.1 Å), indicating that the transformation proceeds predominantly through shape optimization under a limited mass exchange constraint (Figure 3a). Although the A15 phase represents a state of minimized chain stretching, its formation kinetics are markedly slower. To probe the origin of this sluggishness, we monitored the chain conformational evolution (Figure S7a,b) and tracked the intensity difference between the *gauche* and *trans* bands (Δ*I* = *I_G_* – *I_T_*, Figure S7c). At 40 °C, the spectra reveal a distinct reduction in Δ*I* prior to the phase transition to the A15 phase, indicating a transient accumulation of *trans* conformations. This spectral signature implies that the stretching penalty cannot be released immediately; instead, with mass exchange kinetically suppressed, the micelles must undergo energetically costly deformation with unfavorable chain stretching and increased interfacial area to resolve the topological mismatch with the A15 lattice. Consequently, the accumulated stretching stress is released only after a prolonged induction period, as evidenced by the delayed recovery of Δ*I* (increase in *gauche* conformations) observed at 168 h. This significant kinetic lag indicates that a high nucleation barrier emerges, leading to a nucleation-controlled phase transition with characteristics similar to regime I crystallization. In this regime, the scarcity of nucleation events relative to grain growth results in the formation of a few large crystalline grains with specific orientations,^55^ manifested by the spotty peaks observed in 2D SAXS patterns (Figure S8).

In contrast, beginning at 45 °C, temperature-dependent Fourier transform infrared (FTIR) spectroscopy (Figure S9) reveals a shift of the O–H stretching band toward higher wavenumbers, indicating a reduction in the fraction of bound carboxyl groups.^56^ This weakening of core interactions, concomitant with a rapid increase in *gauche* conformations as evidenced by rising Δ*I* (Figure S7c), demonstrates that the system can effectively relieve chain stretching penalties via activated intermicellar mass exchange. Consequently, raising the temperature from 30 to 50 °C causes a contraction in *R̅* of the DDQC phase (Δ*R̅* ≈ −1.7 Å), followed by a discernible radius increase (Δ*R̅* ≈ 0.3 Å) during the isothermal DDQC → σ transition at 50 °C, which is a distinct dimensional adjustment compared to the A15 pathway. These dimensional changes reflect the onset of volume redistribution that allows the system to bypass the high-penalty deformation route, promoting rapid, spatially random nucleation analogous to regime III crystallization, as evidenced by the continuous Debye–Scherrer rings in the 2D SAXS patterns (Figure S10). This mass-exchange-enabled pathway grants access to the thermodynamically favored σ phase, whose stability relies on a broader distribution of particle volumes relative to the A15 phase to optimally balance the thermodynamic competition between interfacial area and chain stretching (Figure 3b). The observation is consistent with the experimental study, indicating that high micellar deformability favors the A15 phase,^45^ as well as theoretical calculations showing that the A15 phase is favored when volume redistribution is restricted, whereas the σ phase becomes the lowest-energy state when volume relaxation through mass exchange is allowed.^57^

This free-energy picture is further supported by the thermal protocols examined in Figure S11. Upon heating, the metastable A15 phase evolves through an A15 + σ coexistence regime and converts into the σ phase after a 10 min hold at 65 °C before isotropizing near 70 °C. In contrast, a preformed σ sample remains in the σ phase during annealing at 40 °C without detectable A15 formation. This sequence is consistent with thermal activation of intermicellar mass exchange, facilitated by the dissociation of hydrogen bonds at elevated temperatures, which enables the system to redistribute micellar volume to access the structurally complex σ phase. In addition, direct cooling from the disordered state to 40 °C produces the σ phase, which becomes better resolved after annealing for 12 h, while prolonged annealing of the DDQC-derived A15 state ultimately leads to the reappearance of σ (Figure S12). Together, these observations are consistent with σ being the thermodynamically favored crystalline phase over A15 at 40 °C, whereas A15 is a long-lived, kinetically trapped metastable FK state accessible through the DDQC → A15 pathway.

### Switching of the DDQC Junction via Core Rigidity

To further manipulate the energy landscape and kinetic stability of the intermediate phases, we employed l-alaninol as a model amine-based guest to modulate the cohesive energy within the micellar core. l-alaninol was selected for its excellent miscibility with the dendritic matrix and its high boiling point (bp = 173–176 °C). As illustrated in Figure 4a, the incorporation of amine guests transforms the hydrogen-bonded soft core into a salt-bridged, rigid network, enabling systematic investigation of the impact of core interaction and core rigidity on phase evolution. FTIR spectroscopy supports the occurrence of proton transfer, evidenced by the disappearance of the carboxylic acid C=O stretching peak and the concurrent appearance of asymmetric and symmetric carboxylate (COO^–^) stretching bands (Figure S13).

**Figure 4:**
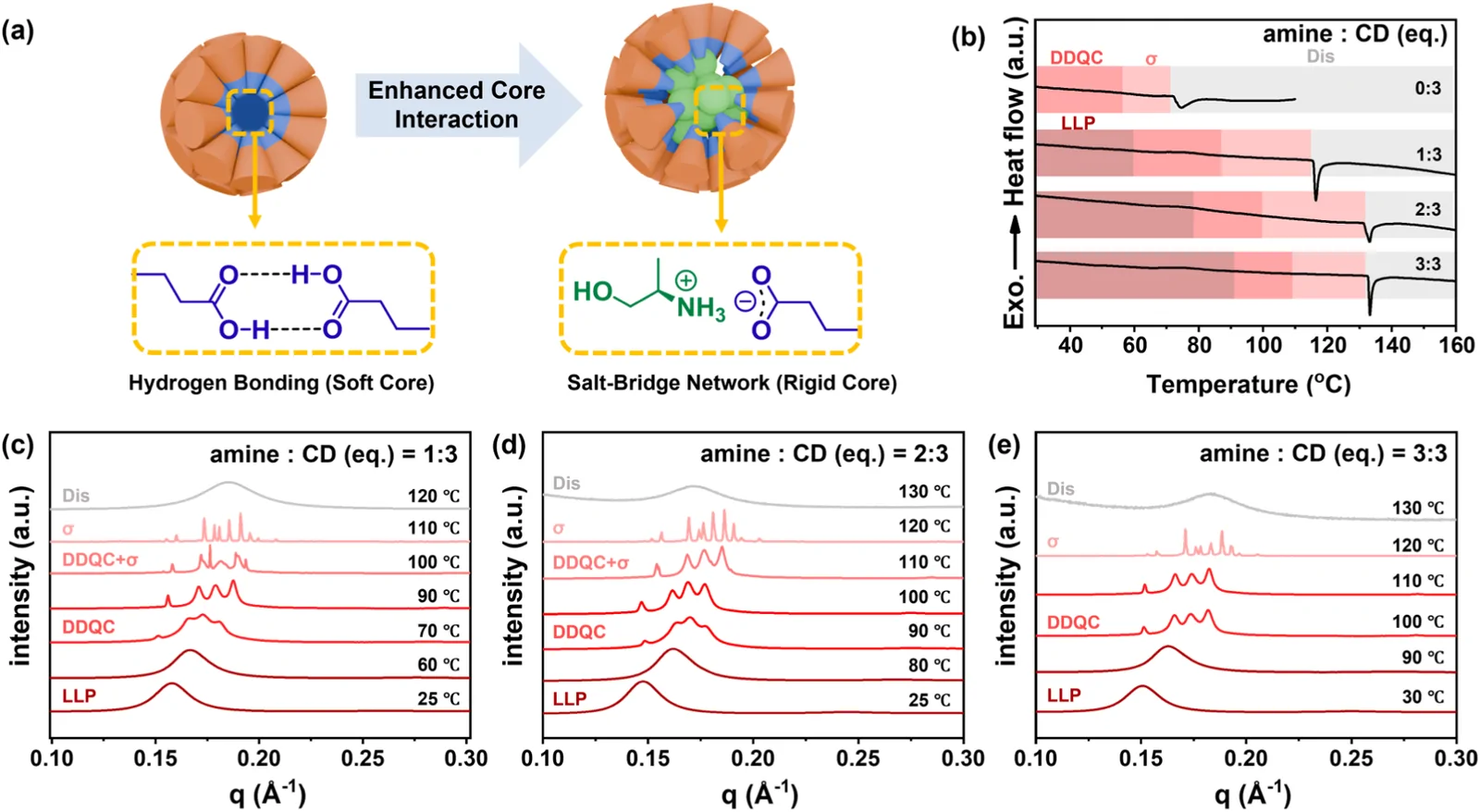
Modulation of phase stability and structural evolution via amine-mediated core interactions. (a) Schematic illustration of the transition from hydrogen-bonded cores to salt-bridge networks. (b) DSC thermograms at different amine-to-**CD** ratios (0:3–3:3). (c–e) *In situ* temperature-dependent SAXS profiles showing the structural evolution of the assemblies with amine-to-**CD** ratios of (c) 1:3, (d) 2:3, and (e) 3:3.

Thermal analysis provides evidence of this stabilization effect. Thermogravimetric analysis (TGA) reveals that the onset of weight loss above 130 °C corresponds to the dissociation of the salt-bridge network and the subsequent volatilization of the released amine guests (Figure S14). Correspondingly, DSC thermograms of the amine-modified **CD** assemblies (Figure 4b) exhibit a systematic elevation in phase transition temperatures with increasing amine content. The isotropization temperature (*T*_i_) shows a marked upward shift as the amine-to-**CD** molar ratio increases from 0:3 to 2:3, before plateauing near 130 °C for the 3:3 complex, consistent with the onset of amine-guest release identified in TGA.

The impact of suppressed mass exchange on structural evolution was further investigated by temperature-dependent SAXS (Figure 4c–e). For the amine-modified **CD** assemblies, the structural evolution originates from a liquid-like packing (LLP) state. This regime corresponds to a dense, disordered arrangement of micelles lacking long-range translational symmetry, serving as the kinetic precursor for the nucleation of complex order.^13^ Upon heating, the system follows an LLP → DDQC → σ phase sequence. As the amine ratio is progressively increased from 1:3 to 3:3, the temperature of the DDQC-to-σ phase transition exhibits a continuous upward shift. This trend suggests that enhanced core interactions increase the thermal persistence of the DDQC phase, effectively trapping the system in this metastable state by impeding the mass redistribution necessary for further evolution into the σ phase.

To elucidate the specific impact of core rigidity on the supramolecular pathway and phase selection, we conducted a stepwise annealing experiment on the 1:3 complex (Figure 5a). The sample was annealed at 60 °C for up to 7 days, followed by sequential isothermal holds at 70 °C (10 h) and 85 °C (3 h). Throughout this extensive thermal history, the DDQC phase exhibited pronounced kinetic stability. Furthermore, the A15 phase remained suppressed, with the system transforming from the DDQC to the σ phase. This finding provides experimental support that the enhanced rigidity suppresses the deformation-driven pathway to the A15 basin. This distinct pathway selectivity is rationalized by the energy landscape depicted in Figure 5b. As detailed earlier, the DDQC → A15 transition is driven predominantly by shape optimization, with limited mass exchange. In the amine-modified system, the transition from flexible hydrogen bonds to a rigid salt-bridge network imposes an energetic penalty along this shape optimization axis. Consequently, the deformation-dependent pathway to A15 becomes unfavorable. The system is thus directed along a trajectory dominated by volume redistribution, leading to the σ phase, where the stability is established via mass exchange—a process effectively activated when elevated temperatures weaken the core interactions. By confining the structural evolution primarily to the DDQC → σ pathway, this study helps clarify the role of micellar rigidity in modulating the competition between distinct FK phases.

**Figure 5:**
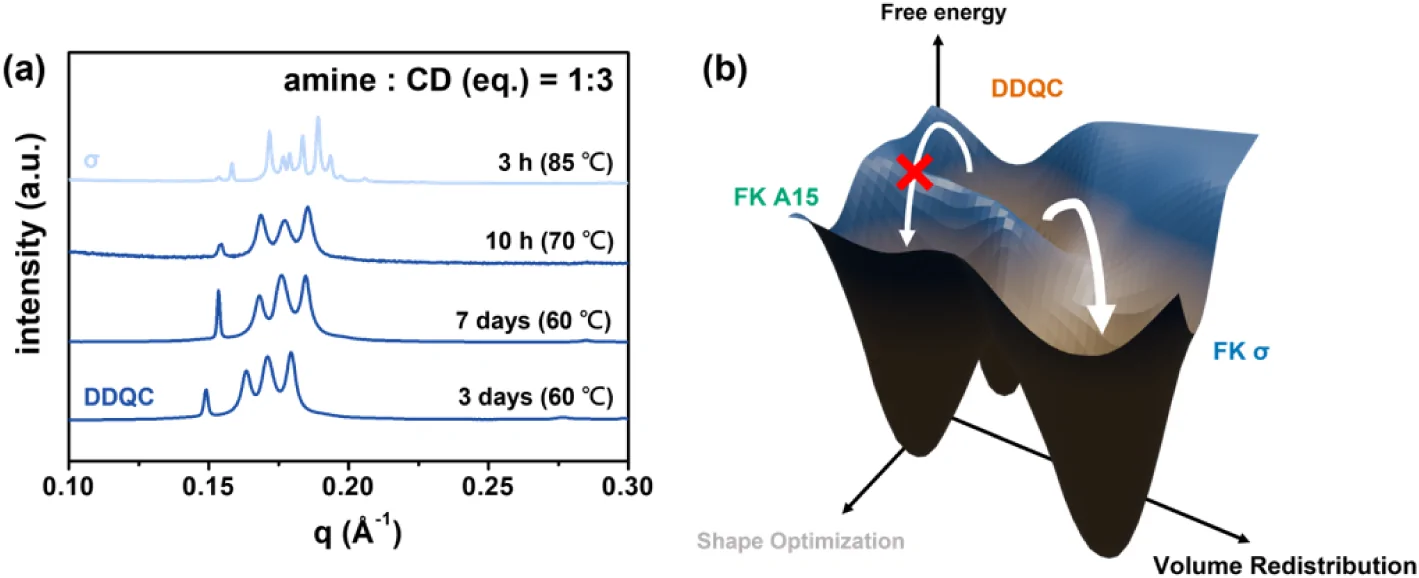
Mechanism of pathway selectivity and suppression of the A15 phase. (a) Time- and temperature-dependent SAXS profiles for the amine-to-**CD** = 1:3 complex during stepwise annealing from 60 to 85 °C. (b) Schematic illustration of the energy landscape of the amine-modified system, showing the suppression of the A15 pathway and the preferential evolution toward the σ phase.

### Chemically Driven Nonequilibrium Transition via VISA

While core interactions modulate phase persistence and core rigidity selects the accessible kinetic pathway, conventional thermal treatments consistently direct the metastable DDQC precursor toward the thermodynamically favored σ phase through a progressive reduction in *R̅* enabled by mass exchange. This behavior limits the structural states accessible through heating and annealing alone. To reverse this structural evolution, we employed VISA as a chemically driven nonequilibrium process. During amine vapor exposure, continuous amine uptake strengthens the core interactions and suppresses intermicellar mass exchange, thereby constraining the redistribution of the existing micellar population. Under this kinetically constrained, composition-changing condition, the system accommodates amine uptake primarily through intramicellar swelling rather than volume redistribution, diverting the assembly from its conventional thermal relaxation pathway.

In this experiment, a pristine **CD** sample was exposed to saturated l-alaninol vapor within a closed chamber at 90 °C. This temperature was selected because intermicellar mass exchange remains thermally accessible during the early stage of amine uptake, allowing the initial ionization of **CD** by the amine to favor the formation of the σ phase. To quantify the uptake kinetics, time-dependent ^1^H NMR analysis (Figure S15) shows that the molar ratio (*n*_amine_/*n*_CD_) increases monotonically and reaches saturation (≈1.2) within 5 min, with no further increase at 6 min. Structural evolution was monitored using *in situ* time-resolved SAXS (Figure 6a). Within the initial 20 s, the profiles exhibit broad scattering features characteristic of a disordered state, consistent with the behavior of neat **CD** at 90 °C. As amine uptake progresses and the core interactions strengthen, Bragg reflections associated with the σ phase emerge at 40 s. Upon approaching saturation after 5 min, the scattering pattern transforms into the distinctive signature of the DDQC phase. The corresponding peak positions and lattice indices are listed in Figure S16 and Table S5. As a complementary control, a premixed **CD**/l-alaninol sample with amine:**CD** = 1.2:1 was heated to the disordered state and directly cooled to 90 °C, where the σ phase formed without detectable DDQC reflections (Figure S17). This result is consistent with DDQC being a metastable state at this fixed composition and shows that, at 90 °C, DDQC is accessed through the nonequilibrium VISA pathway. Quantitative analysis of these profiles reveals a continuous increase in *R̅* (Figure 6b and Table S6), marking a direct reversal of the shrinkage observed during thermal annealing. This suggests that the structural evolution is driven by enhanced core interactions and micellar swelling. According to the diblock foam model (DFM), the free energy of the system is governed by a balance between interfacial energy (∝1/*R*_0_) and chain-stretching energy $\left(\propto R_{0}^{2}\right)$ , where *R*_0_ denotes the equal-volume spherical radius, defined from the mean cell volume, corresponding to the experimentally estimated *R̅* used in this study.^57^ In this framework, the observed increase in *R̅* during the VISA process is consistent with an enhanced chain-stretching penalty. This interpretation is further supported by Raman spectroscopy (Figure S18). Tracking the relative intensity difference between the *gauche* and *trans* conformers reveals a decrease over time, indicating an increased population of extended, trans-rich chain conformations. Consistently, the average particle radius increases from *R̅*_σ_ = 23.4 Å to *R̅_DDQC_* = 26.1 Å during the σ-to-DDQC transition. This correlation suggests that VISA-induced micellar swelling is accompanied by alkyl-chain extension, promoting the transformation from the compact σ phase to the swollen, metastable DDQC state.

**Figure 6:**
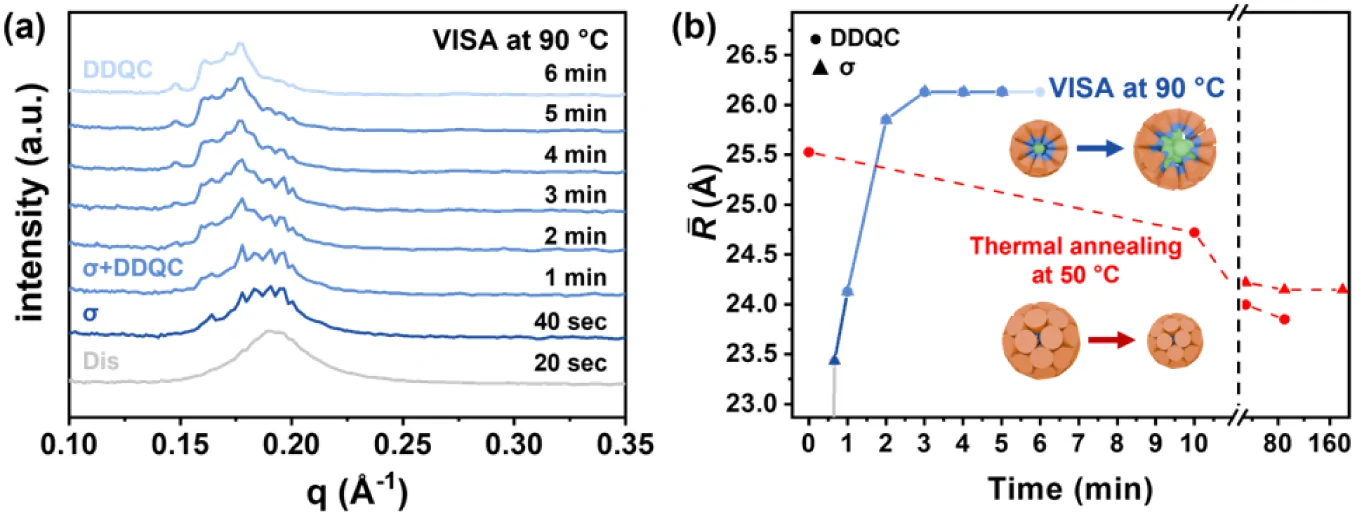
Isothermal phase transitions driven by VISA. (a) *In situ* time-resolved SAXS profiles monitoring the structural evolution of a pristine **CD** sample exposed to saturated l-alaninol vapor at 90 °C. (b) Time evolution of the average particle radius (*R̅*), comparing VISA-induced swelling at 90 °C (blue) with the thermal relaxation during annealing at 50 °C (red). The schematic illustrates the opposite evolution of micellar size under VISA and thermal annealing conditions.

This transition demonstrates that chemical modulation of core interactions can induce micellar swelling, enabling the system to access a DDQC state and counteract the intrinsic thermal relaxation pathway. However, due to the low crystallinity observed at 90 °C, the VISA temperature was further increased to 115 °C. Under this condition, time-dependent ^1^H NMR analysis shows that amine uptake proceeds more slowly at 115 °C. Nevertheless, the molar ratio (*n*_amine_/*n*_CD_) approaches 1.2 within 6 min, comparable to the final uptake level observed at 90 °C (Figure S19). After completion of the vapor-exposure step, the l-alaninol source was removed, and the sample was subsequently cooled without further exposure to amine vapor. ^1^H NMR analysis of the VISA-treated sample before and after cooling showed comparable amine:**CD** ratios of approximately 1.2:1, indicating that the subsequent cooling process occurred under an approximately fixed amine-content condition (Figure S20). As shown in Figure 7a, during VISA at 115 °C, the elevated temperature allows the system to form the σ phase with enhanced crystallinity compared with that obtained during VISA at 90 °C. The σ phase remains predominant upon cooling to 90 °C, although its reflections become noticeably broader than those observed at 115 °C. Upon further cooling to 60 and 40 °C, the characteristic σ reflections progressively broaden and partially disappear, accompanied by the emergence of DDQC reflections. At 25 °C, the initially broadened DDQC scattering feature gradually evolves into a pronounced (12100) reflection after aging for 1 day. To determine whether this cooling-induced transition is unique to the VISA-treated sample, a premixed **CD**/l-alaninol sample with the same final composition of amine:**CD** = 1.2:1 was examined under the same cooling protocol. Although the premixed sample also underwent a σ-to-DDQC transition, the σ phase persisted to a lower temperature than in the VISA-treated sample (Figure S21). This comparison indicates that VISA does more than simply establish the final amine content; the nonequilibrium vapor-uptake process kinetically preconditions the σ precursor into a less-equilibrated structural state, thereby promoting the subsequent cooling-induced σ-to-DDQC transition. Upon cooling, the salt-bridge-reinforced micellar cores increasingly restrict intermicellar mass exchange such that micellar swelling cannot be fully accommodated through the volume redistribution required to preserve the σ lattice. This kinetic restriction prevents the system from maintaining the micellar volume distribution preferred by the σ lattice during cooling, thereby redirecting the assembly toward a metastable DDQC state. This interpretation is further supported by Raman spectroscopy, together with the SAXS-derived micellar dimensions (Figure S22). Raman spectra show an increased contribution of trans-rich chain conformations after cooling, indicating that the alkyl chains become more extended. Consistently, the average particle radius increases from R̅_σ_ = 25.2 Å to *R̅_DDQC_* = 28.4 Å during the cooling-induced σ-to-DDQC transition. This agreement between Raman and SAXS indicates that the transition is accompanied by micellar swelling and chain extension. Importantly, because the σ phase established at 115 °C has high crystallinity, this swelling-induced σ-to-DDQC transformation is expected to require substantial structural reorganization, consistent with a relatively high nucleation barrier. This effectively suppresses excessive random nucleation and preserves a growth environment conducive to the emergence of the 12-fold diffraction pattern. The importance of this high-crystallinity precursor is further corroborated by the temperature-dependent SAXS patterns in

**Figure 7:**
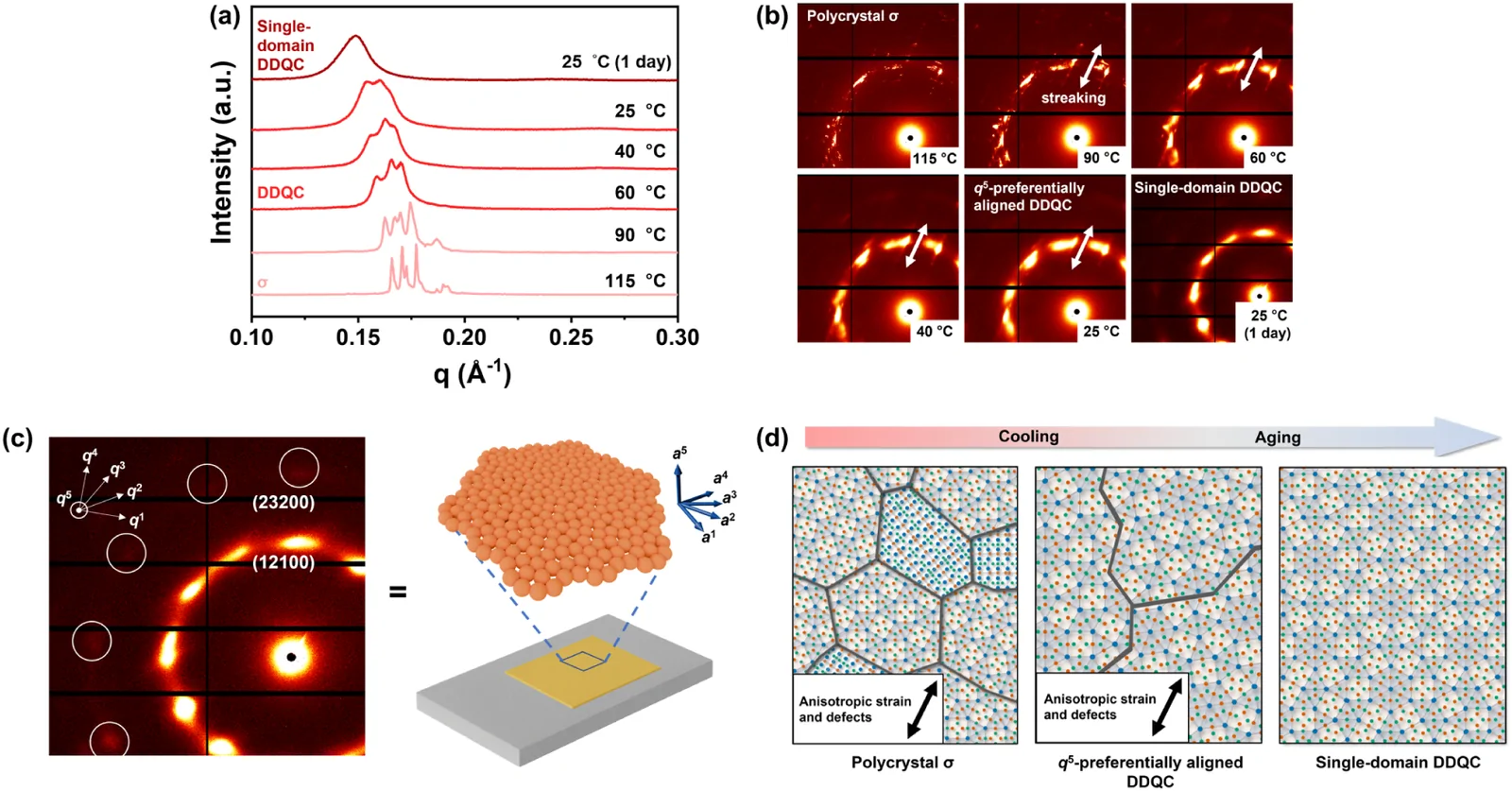
Macroscopic alignment and thermal evolution of the face-on DDQC. (a) 1D SAXS profiles showing the σ-to-DDQC transition under cooling. (b) Temperature-dependent 2D SAXS patterns during cooling and subsequent aging at 25 °C. (c) Indexed 2D SAXS pattern and schematic illustration of the face-on single-domain DDQC. (d) Proposed mechanism illustrating how anisotropic reconstruction and defect relaxation during cooling and aging facilitate in-plane quasiperiodic reorganization toward face-on single-domain DDQC formation.

Figure S23, which show that increasing the VISA temperature enhances structural ordering, thereby enabling the formation of the DDQC pattern upon subsequent cooling. Upon further cooling from 90 to 40 °C, the temperature-dependent 2D SAXS patterns show a gradual transition from polydomain σ scattering to DDQC reflections, accompanied by streaking of the emerging reflections along the substrate-parallel direction. At 25 °C, the 2D SAXS pattern displays a *q* ^5^-preferentially aligned DDQC with 12-fold diffraction spots. After aging at 25 °C for 1 day, the streaking disappears, and the 2D SAXS pattern sharpens into well-defined 12-fold spots (Figure 7b). As shown in Figure 7c, the VISA-directed DDQC film exhibits two concentric sets of diffraction spots with 12-fold rotational symmetry, which are assigned to the (12100) and (23200) reflections of the DDQC lattice (see Figure S24 and Table S7 for detailed indexing). Notably, these highly resolved spots were obtained using a macroscopic X-ray beam size of 1.5 × 1.0 mm. The absence of continuous powder rings over such a large irradiated area provides strong evidence that the quasiperiodic order extends over macroscopic length scales, yielding a macroscopic single-domain DDQC with a face-on orientation. The reproducibility of this face-on DDQC formation was further confirmed using four independently prepared samples, all of which exhibited characteristic 12-fold diffraction features under the same VISA conditions (Figure S25). Spatially resolved 2D SAXS mapping further shows well-defined 12-fold diffraction patterns with nearly identical azimuthal orientations across six positions spanning nearly the entire crystalline domain, confirming a common orientational registry over the mapped millimeter-scale area (Figure S26a,b). In addition, 2D SAXS mapping at four positions within each of two spatially separated regions of a larger VISA-treated sample with dimensions of approximately 10 × 5 mm reveals nearly identical azimuthal orientations within each region but distinct orientations between the two regions, demonstrating the reproducible formation of highly oriented DDQC grains at different locations within the sample (Figure S26c). The streaking observed in the 2D SAXS patterns indicates anisotropic structural reconstruction during the σ → DDQC transition. This anisotropic scattering feature reveals a limited correlation length along the substrate-parallel direction in real space and may also reflect residual internal strain in the intermediate state,^58–60^ arising from micellar swelling and the associated chain extension during cooling. This *q* ^5^-aligned configuration enhances the lateral extent of the quasiperiodic plane, thereby providing greater structural flexibility for redistributing mismatch-related strain.^61^ Such stress redistribution may facilitate the coalescence of neighboring quasicrystalline regions by promoting grain rotation toward reduced misorientation and phason-assisted relaxation during the final stage of ordering (Figure 7d).^62,63^

## Conclusion

In conclusion, this study establishes the DDQC as a metastable kinetic junction within the complex energy landscape of dendritic self-assembly. Using time-resolved SAXS to trace the structural evolution, we revealed that pathway selection from this DDQC junction toward either the A15 or the σ phase is governed by a controllable competition between shape optimization and volume redistribution, regulated by the accessibility of intermicellar mass exchange and the rigidity of the micellar cores. We further demonstrated that this landscape can be programmed through core engineering. The introduction of amine-mediated salt bridges rigidifies the micellar cores, imposing an energy barrier on the deformation-driven A15 pathway while preserving access to the mass-exchange-mediated σ phase at elevated temperatures. This kinetic control was further extended through VISA, a chemically driven nonequilibrium process in which composition-changing amine uptake kinetically conditions the σ precursor, while subsequent cooling provides access to a metastable DDQC state. The high nucleation barrier associated with this nonequilibrium pathway grants access to a nucleation-controlled regime, thereby enabling the formation of a macroscopically aligned, face-on single-domain DDQC. Ultimately, our findings establish core engineering as a general strategy for navigating rugged energy landscapes. By transforming metastability from an uncontrolled kinetic trap into a tunable design element, this work provides a framework for the rational design of reconfigurable materials with programmable complexity.

## Supplementary Material



## Notes

Synthetic procedures; ^1^H and^13^C NMR and mass spectra of **CD**; structural-analysis calculations; Miller-index assignments and SAXS patterns of the A15, σ, and DDQC phases; preparation of amine-modified **CD** assemblies; VISA procedures; and DSC, Raman, FTIR, and TGA characterization (PDF)
