# Supplementary material for: Nonequilibrium Navigation of a Rugged Energy Landscape Enables the Formation of Face-On Single-Domain Dodecagonal Quasicrystals
Source: J Am Chem Soc. 2026 Sep 30;148(42):45457–67. doi: 10.1021/jacs.6c09814 (PMC13721164; doi:10.1021/jacs.6c09814)
Supplement: Supplementary file 1 [file ja6c09814_si_001.pdf]

Supporting Information

Non-Equilibrium Navigation of a Rugged Energy Landscape Enables the Formation of Face-On Single-Domain Dodecagonal Quasicrystals

Mayumi Egashira[a], Jing-Wei Chen[a], Mao-Yan Wang[a], Chun-Jen Su[b], Yi-Qi Yeh[b], Wei-Tsung Chuang[b]*, Chien-Lung Wang[a]*

[a]Department of Chemistry, National Taiwan University, No. 1, Sec. 4, Roosevelt Rd, Taipei 10617, Taiwan

[b]National Synchrotron Radiation Research Center, 101 Hsin-Ann Road, Hsinchu 30076, Taiwan

All authors have given approval to the final version of the manuscript.

*** Corresponding Author**

Email: weitsung@nsrrc.org.tw

*** Corresponding Author**

Email: kclwang@ntu.edu.tw

Supporting Information

Table of Contents

Table of Contents 1

Experimental Section 2

1. Synthetic Procedures of the carboxyl-functionalized dendron (CD) 2

2. Experimental Details 3

Material Characterization 10

1. NMR and MS Spectra of Products 10

2. Characterization of the CD 12

3. Characterization of Amine-Modified CD Assemblies 22

References 35

Experimental Section

1. Synthetic Procedures of the carboxyl-functionalized dendron (CD)

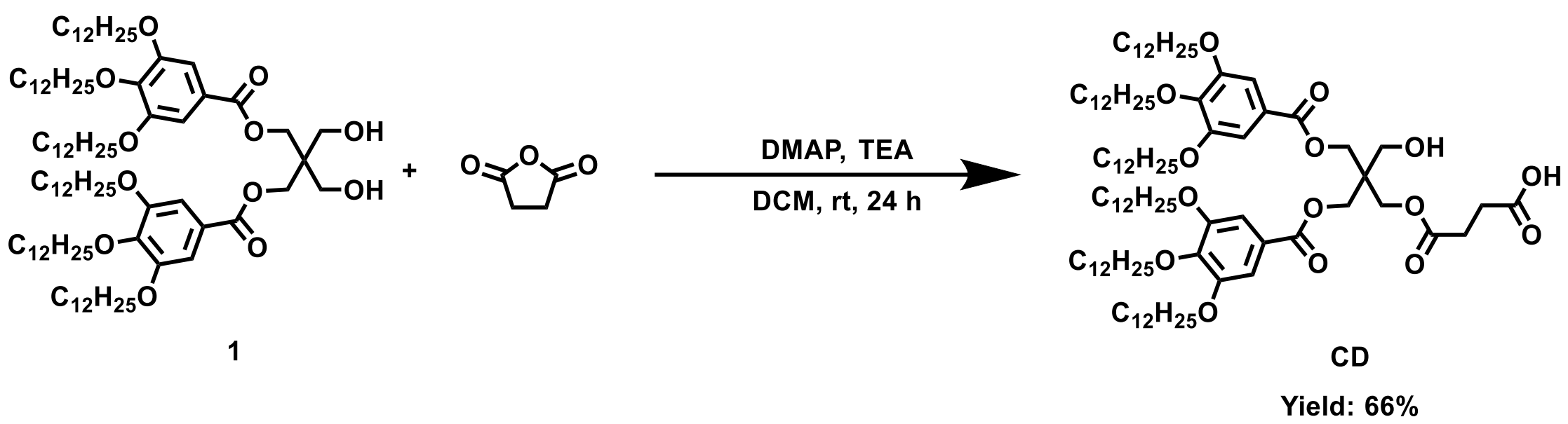

Scheme S1. The synthetic route of the **CD**.

Compound 1 was prepared following a previously reported method.[1] As shown in **Scheme S1**, **CD** was synthesized from **compound 1** via succinic anhydride coupling. **Compound 1** (1 equiv., 0.69 mmol, 1000 mg), succinic anhydride (1.2 equiv., 0.83 mmol, 83 mg), 4-(dimethylamino)pyridine (DMAP, 0.5 equiv., 0.35 mmol, 43 mg), triethylamine (TEA, 2.0 equiv., 1.38 mmol, 192 μL) were dissolved in dichloromethane (DCM, 50 mL). The reaction mixture was stirred at rt for 24 h under N_2(g). After completion, distilled water (200 mL) was added, and the mixture was extracted with dichloromethane (50 mL × 6). The organic layers were dried over anhydrous $MgSO_4$, filtered and the solvent was removed under reduced pressure. The residue was further purified by silica gel column chromatography (EtOAc/hexane = 1:4 followed by MeOH/EtOAc = 1:19) and concentrated *in vacuo* to afford **CD** as a white solid (702 mg, 66% yield). ^{1}H NMR ($CDCl_3$, 400 MHz): δ = 7.21 (s, 4H), 4.47 (d, *J* = 12.0 Hz, 2H), 4.41 (d, *J* =12.0 Hz, 2H), 4.33 (s, 2H), 4.03-3.96 (m, 12H), 3.65 (s, 2H), 2.66 (s, 4H), 1.83-1.70 (m, 12H), 1.48-1.43 (m, 12H), 1.38-1.26 (m, 96H), 0.88 (t, *J* = 8.0 Hz, 18H) ppm. ^{13}C NMR ($CDCl_3$, 125 MHz): δ = 175.1, 172.2, 166.6, 153.1, 143.1, 124.0, 108.3, 73.3, 69.4, 62.9, 60.9, 45.0, 32.1, 30.5, 29.90, 29.89, 29.87, 29.85, 29.82, 29.81,

29.74, 29.61, 29.55, 29.53, 29.50, 29.14, 28.66, 26.28, 26.22, 22.8, 14.3. HRMS (ESI-QTOF): m/z $[M+H]^+$ calcd for $C_{95}H_{169}O_{15}$ 1550.2456, found 1550.2420.

2. Experimental Details

1. Materials and Characterization Techniques.

All chemicals were used without further purification. The chemicals and their purities are listed as follows: 1-bromododecane (Nova Materials, 98.5%), methyl gallate (Nova Materials, 98.5%), potassium carbonate (Nova Materials, 99%), potassium hydroxide (SHOWA, >85%), pentaerythritol (Thermo Scientific, 98%), 4-(dimethylamino)pyridine (Nova Materials, 98%), 1-(3-dimethylaminopropyl)-3-ethyl carbodiimide hydrochloride (Nova Materials, 97%), succinic anhydride (Nova Materials, 99%), triethylamine (ECHO, >98%), (*S*)-(+)-2-amino-1-propanol (Nova Materials, 98%). **Nuclear magnetic resonance (NMR):** ^{1}H and ^{13}C NMR measurements were performed using a Bruker AVIII HD 400 MHz NMR (400 MHz for ^{1}H) and Bruker AVIII 500 MHz FT-NMR (125 MHz for ^{13}C) with $CDCl_3$ as the deuterated solvent. Approximately 2–5 mg of each sample was used for data acquisition. **Mass spectrometry (MS):** High-resolution mass spectra (HRMS) were recorded in positive-ion electrospray ionization (ESI) mode using a Bruker micrOTOF-QII. **Small-angle X-ray scattering (SAXS):** SAXS experiments were conducted at the TPS 13A and TLS 23A1 beamlines of the National Synchrotron Radiation Research Center (NSRRC), Taiwan. The TPS 13A beamline utilized X-rays with a wavelength (λ) of 0.8265 Å and a focused beam size of 400 × 200 μm^2. The TLS 23A1 beamline utilized X-rays with a wavelength (λ) of 0.8265 Å and a focused beam size of 1.5 × 1.0 mm^2. Data were collected over a scattering vector (q) range of 0.004–0.45 Å^{-1}. For sample preparation, approximately 1 mg of the specimen was encapsulated between two layers

of heat-resistant Kapton tape. **Differential scanning calorimetry (DSC):** DSC measurements were performed using a TA Instruments Discovery DSC 2500. For each analysis, approximately 1–3 mg of the sample was encapsulated in standard aluminum pans. The thermograms were recorded at a heating and cooling rate of 10 °C/min under a nitrogen atmosphere. **Fourier transform infrared spectroscopy (FTIR):** Infrared spectra were recorded using a Shimadzu IRSpirit spectrophotometer equipped with an attenuated total reflectance (ATR) accessory. For measurement, a small aliquot (1–3 mg) of the sample was placed directly onto the crystal surface. Each spectrum was acquired by accumulating 32 scans. **Raman spectra:** Raman spectra were acquired using a custom-built micro-Raman system. The samples were excited by a 532 nm Cobolt Samba laser. The resulting scattering signals were collected via a Southport Jademat NM system integrated with a Kymera 328i B1 Andor spectrometer. **Thermogravimetric analysis (TGA):** Thermal stability was evaluated using a TA Instruments Q500 thermogravimetric analyzer. Samples were heated from room temperature to 600 °C at a scan rate of 10 °C/min under nitrogen flow.

2. Calculation for structural analysis

Calculation of diffraction peak positions (q_{calc})

For the dodecagonal quasicrystal (DDQC), which lacks translational periodicity, theoretical peak positions and characteristic q ratios were derived from the calculated diffraction pattern of the DDQC model as reported by Zeng et al.[2]

For the σ phase, the calculated peak positions were determined using:

$$q_{calc} = 2\pi\sqrt{\frac{h^2 + k^2}{a^2} + \frac{l^2}{c^2}}$$

For the Frank-Kasper A15 phase, the calculated peak positions were determined using:

$$q_{calc} = 2\pi \sqrt{\frac{h^2 + k^2 + l^2}{a^2}}$$

where *h, k, l* are the Miller indices, and *a* and *c* are the lattice parameters.

Peak indexing was validated by comparing the experimental q ratios (q_{obs}/q^*, where q^* is the primary peak position) with the theoretical ratios inherent to these crystal symmetries. The indexed reflections, observed absolute positions (q_{obs}), and the corresponding comparison of ratios are tabulated in **Table S1** (DDQC), **Table S2** (A15) and **Table S3** (σ).

Calculation of Average Particle Radius ($\bar{R}$).

For the A15 phase, the unit cell contains 8 micelles. The lattice parameter a was determined from the primary scattering peak $q_{(200)}$ using the relation $a = 4\pi/q_{(200)}$. $\bar{R}_{A15}$ is given by:

$$\bar{R}_{A15} = \left(\frac{3}{4\pi} \times \frac{a^3}{8}\right)^{1/3} = \frac{6^{\frac{1}{3}}\pi^{\frac{2}{3}}}{q_{(200)}}$$

For the σ phase, the unit cell contains 30 micelles. The unit cell volume is given by $V_{cell} = a^2c$. The lattice parameters were determined from the scattering peaks as follows: the parameter c was calculated from the $q_{(002)}$ peak using $c = 4\pi/q_{(002)}$, and the parameter a was calculated from the $q_{(330)}$ peak using $a = 6\pi\sqrt{2}/q_{(330)}$. Based on these calculations, the experimental lattice parameter ratio was determined to be $c/a = 0.526$. Finally, $\bar{R}_{\sigma}$ is derived as:

$$\bar{R}_{\sigma} = \left(\frac{3}{4\pi} \times \frac{a^2c}{30}\right)^{1/3} = \frac{2\pi^{\frac{2}{3}}}{5^{\frac{1}{3}}\left(\frac{c}{a}\right)^{\frac{2}{3}} q_{(002)}}$$

For the DDQC phase, unlike periodic crystals, the DDQC lacks a finite unit cell, precluding an exact calculation based on lattice parameters. However, due to its strong structural kinship with the σ phase, its particle radius can be reliably approximated using the σ phase geometry. Following the methodology reported by Lindsay et al., the average particle radius was approximated using the σ phase geometry by adopting the experimental aspect ratio of $c/a = 0.526$ from the σ phase and substituting the $q_{(002)}$ reflection of σ phase with the $q_{(00002)}$ reflection of DDQC phase.[3] The average radius is thus calculated as:

$$\bar{R}_{\mathrm{DDQC}} = \frac{2\pi^{\frac{2}{3}}}{5^{\frac{1}{3}}\,(0.526)^{\frac{2}{3}}\, q_{(00002)}}$$

3. Preparation of amine-modified CD assemblies

L-alaninol (10 mg) and **CD** (30 mg) were separately dissolved in 1 mL each of dichloromethane (DCM) to prepare the stock solutions. To obtain the supramolecular complexes with varying amine concentrations, specific volumes of the L-alaninol stock solution (48, 97, and 145 μL, respectively) were added to a fixed volume of the **CD** stock solution (1 mL) to target amine-to-**CD** molar ratios of 1:3, 2:3, and 3:3. After mixing, the solvent was evaporated under reduced pressure. The samples were then dried *in vacuo* to constant weight prior to structural characterization.

4. *In situ* Vapor-induced self-assembly (VISA) SAXS Experiments

To investigate the structural evolution driven by amine uptake, a vapor annealing chamber was employed (**Scheme S2**). Approximately 2.0 mg of the pristine **CD** sample was loaded onto a Kapton tape substrate, which was subsequently mounted on a hollow metal disk to allow for transmission X-ray measurements. The assembly was then placed inside the chamber equipped with X-ray transparent Kapton windows. Prior to vapor exposure, the heating stage was heated to 90 °C and maintained at this temperature to ensure that the sample was in a completely molten state. Once the temperature stabilized, saturated amine vapor was introduced into the chamber, and the structural evolution was monitored *in situ* under these isothermal conditions.

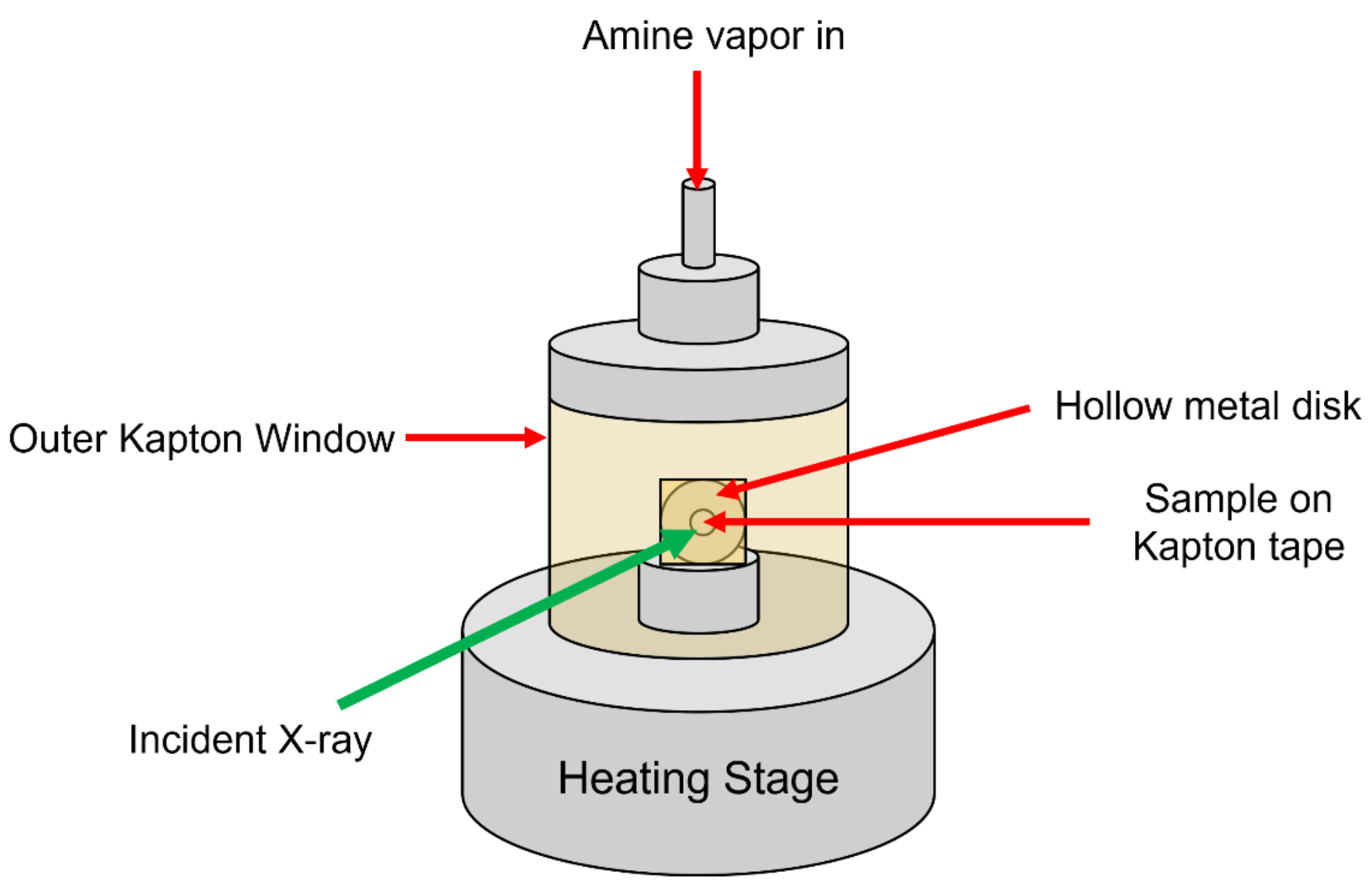

Scheme S2. Schematic illustration of the *in situ* vapor-induced self-assembly (VISA) SAXS chamber.

5. Preparation of single-domain DDQC via VISA

A pristine **CD** sample (2.0 mg) was deposited onto a Kapton tape substrate and placed on a temperature-controlled heating stage. The sample was heated to 115 °C and maintained at this temperature until complete melting was achieved and bubbles disappeared. Subsequently, 100 μL of L-alaninol was introduced adjacent to the sample, and the system was immediately covered with a Petri dish (diameter: 10 cm) to establish a saturated amine vapor environment (**Scheme S3**). The sample was exposed to the vapor for 6 min under isothermal conditions. After vapor treatment, the cover was removed to allow excess amine to dissipate, and the sample was allowed to cool to 25 °C.

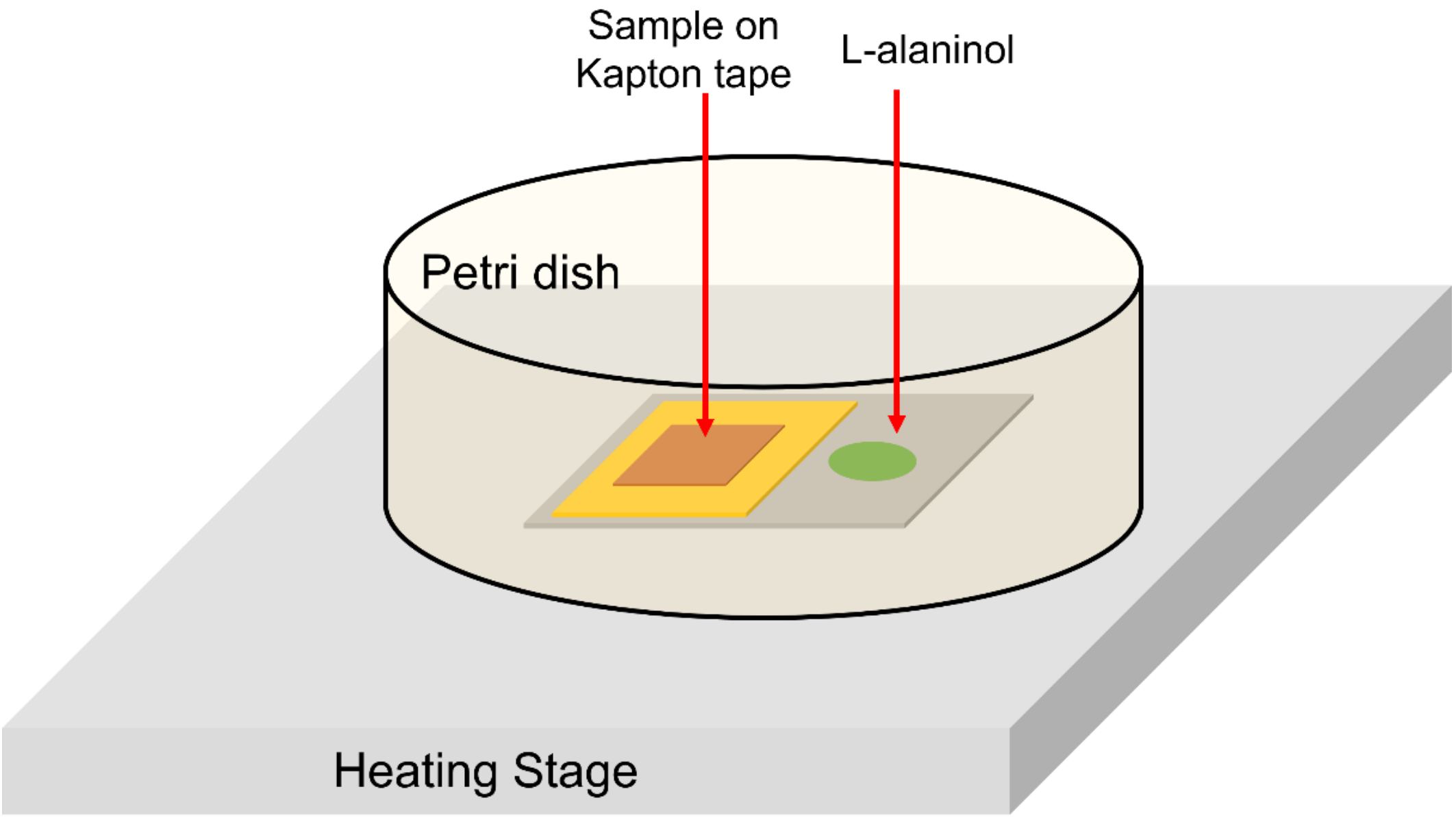

Scheme S3. Experimental configuration for vapor-induced self-assembly (VISA) for the preparation of single-domain DDQC.

Material Characterization

1. NMR and MS Spectra of Products

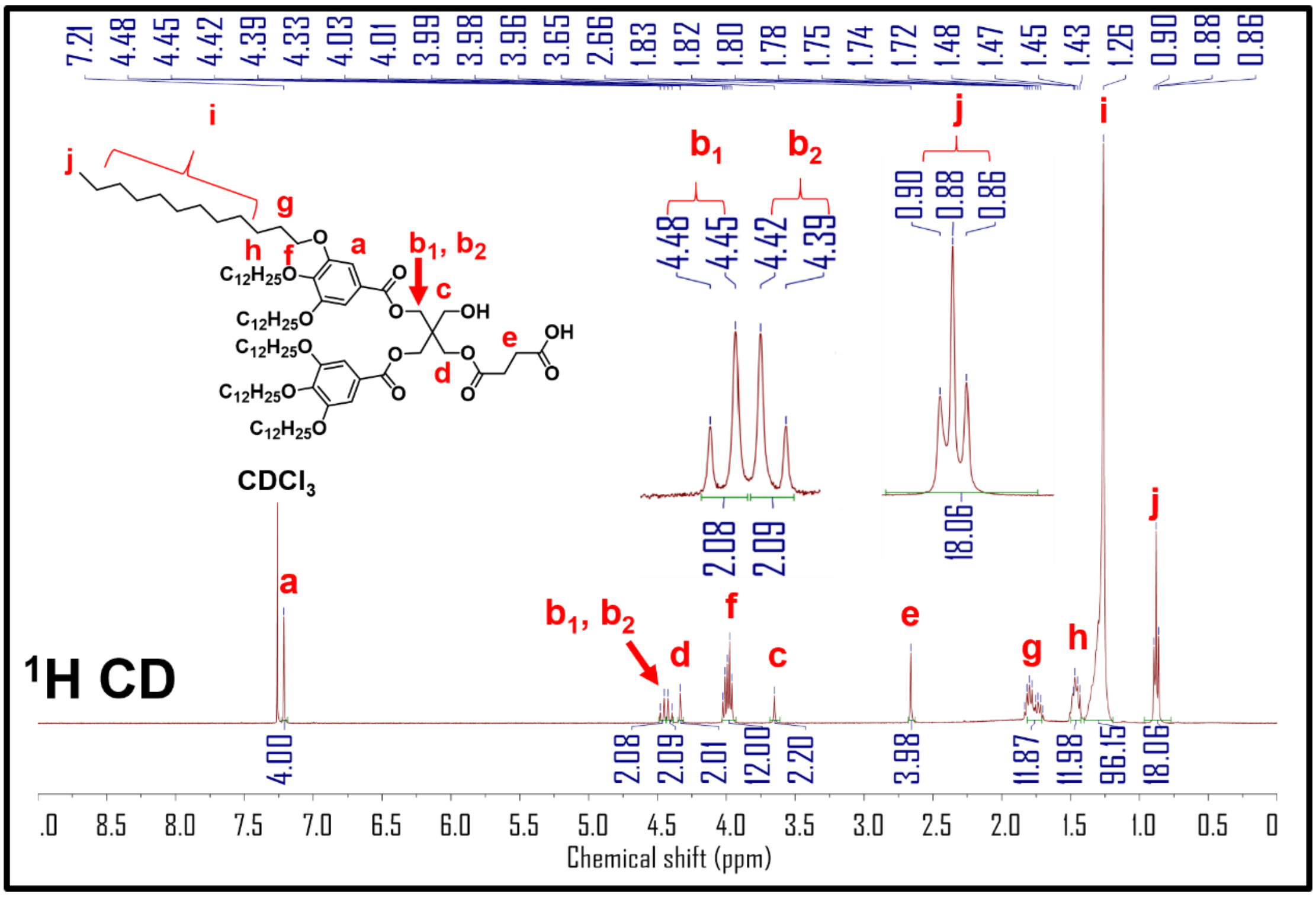

Figure S1. ^{1}H NMR spectrum of **CD** (400 MHz in $CDCl_3$).

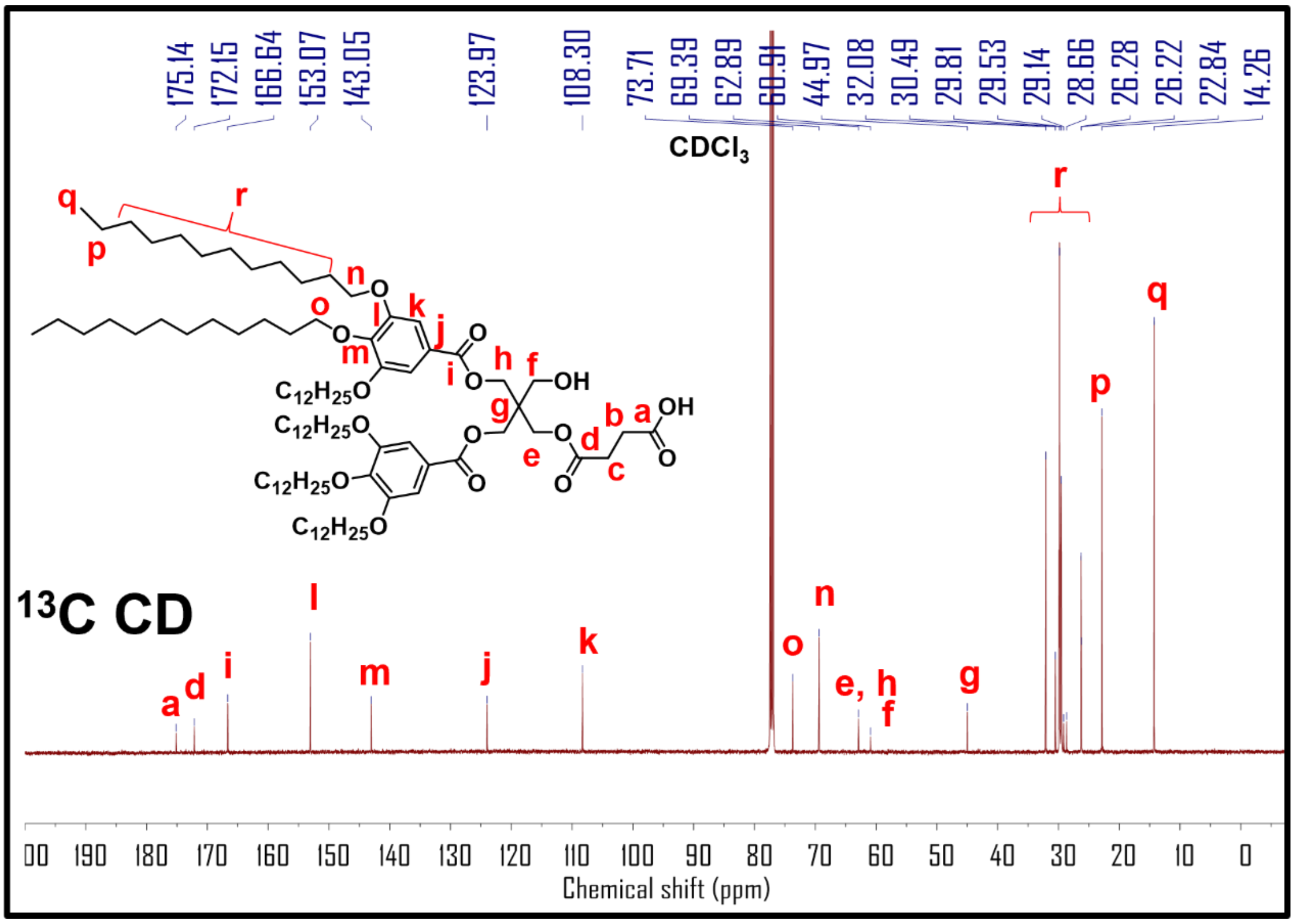

Figure S2. ^{13}C NMR spectrum of **CD** (125 MHz in $CDCl_3$).

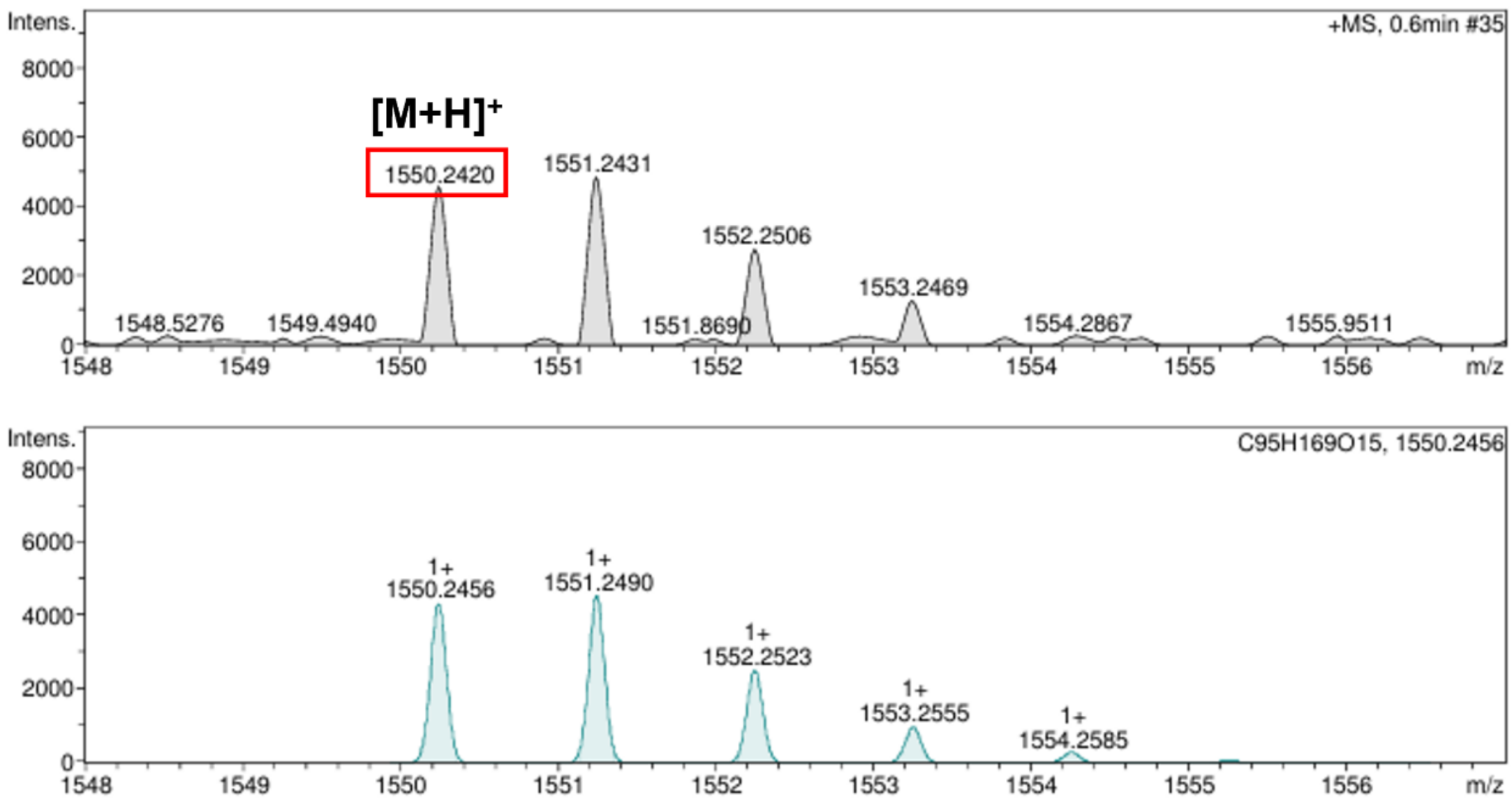

Figure S3. ESI-MS spectrum of **CD**.

2. Characterization of the CD

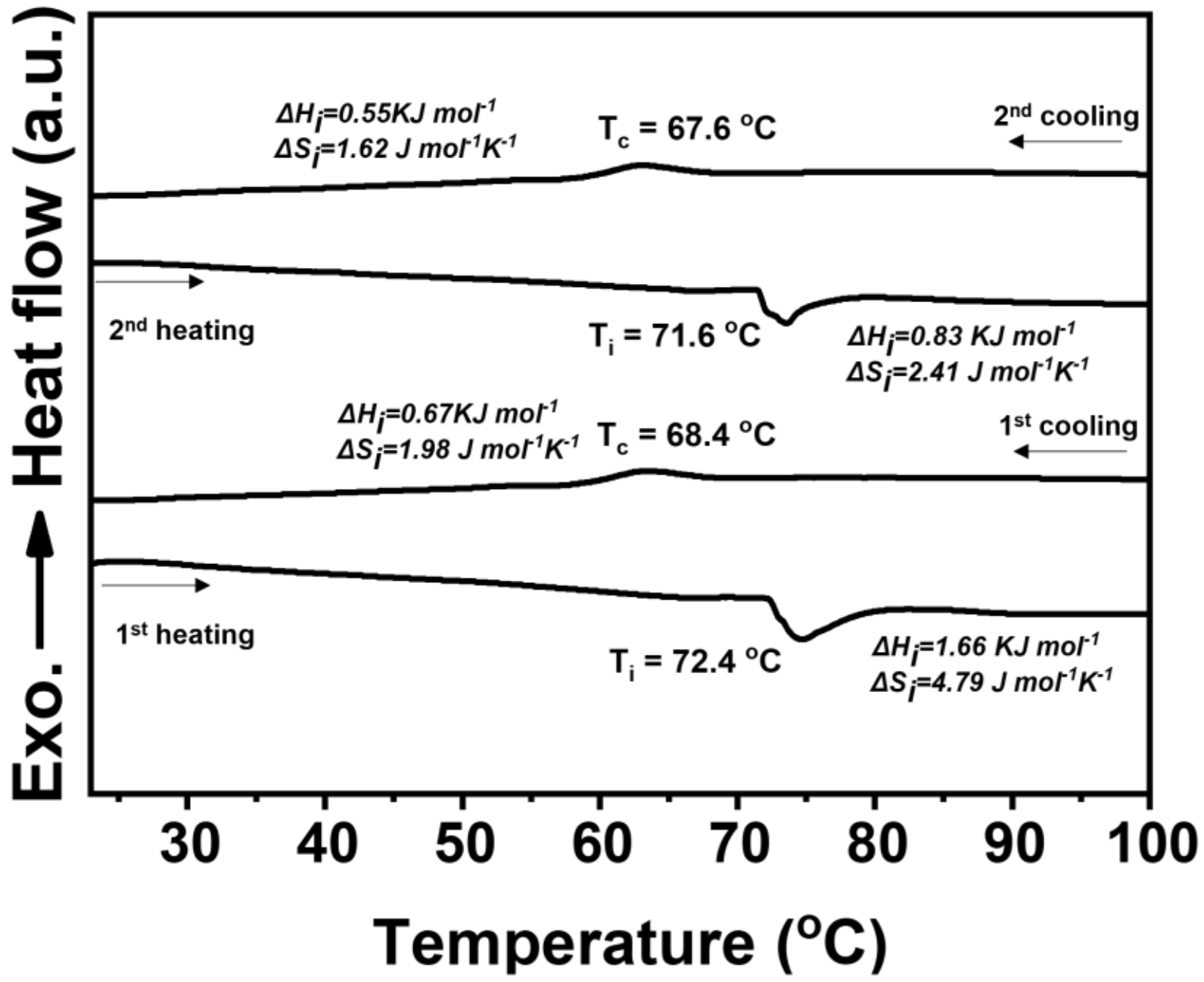

Figure S4. DSC thermograms of the **CD** recorded at a heating and cooling rate of 10 °C min^{-1}.

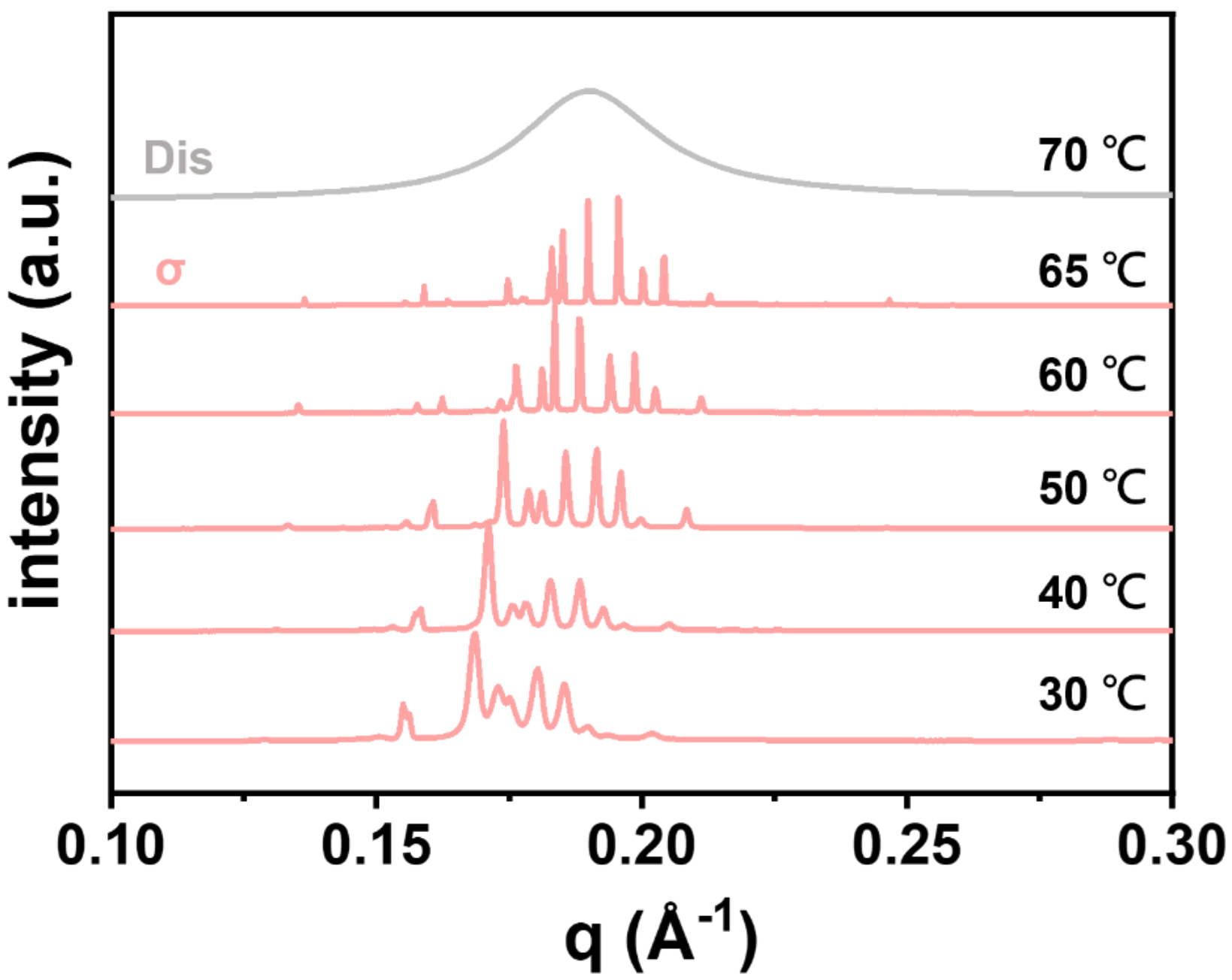

Figure S5. *In situ* temperature-dependent SAXS profiles of **CD** assemblies during the first cooling scan.

Table S1. Experimental and calculated diffraction data for the DDQC phase. The diffraction indices correspond to the diffraction peaks labeled in **Figure 2c**.

**indices (h_1 h_2 h_3 h_4 h_5)**	q_{calc} **ratio**	q_{obs} **(Å^{-1})**	q_{obs} **ratio**	$\Delta q/q_{calc}$ **(%)**
0 0 0 0 2	1.00	0.1543	1.000	0.00
1 2 1 0 0	1.08	0.1699	1.101	1.94
1 0 1 0 2	1.12	0.1775	1.150	2.68
1 2 1 0 1	1.19	0.1862	1.207	1.43
1 2 2 1 0	1.52	0.2395	1.552	2.11
1 2 1 0 3	1.85	0.2871	1.861	0.59
2 2 2 0 2	1.87	-	-	-
2 3 2 0 0	1.87	0.2955	1.915	2.41
2 3 2 0 1	1.93	-	-	-
0 0 0 0 4	2.00	0.3086	2.000	0.00
1 3 3 1 0	2.08	-	-	-
2 4 2 0 0	2.15	0.3377	2.189	1.81
1 2 1 0 4	2.27	0.3522	2.283	0.57

Table S2. Experimental and calculated diffraction data for the A15 phase at 40 °C. The Miller indices correspond to the diffraction peaks labeled in **Figure 2d**.

**Miller indices (h k l)**	q_{calc} **ratio**	q_{obs} **(Å^{-1})**	q_{obs} **ratio**	$\Delta q/q_{calc}$ **(%)**
1 1 0	1.000	0.1117	1.000	0.00
2 0 0	1.414	0.1578	1.413	-0.09
2 1 0	1.581	0.1763	1.579	-0.14
2 1 1	1.732	0.1933	1.731	-0.09
2 2 0	2.000	0.2231	1.998	-0.12
3 1 0	2.236	0.2495	2.234	-0.08
2 2 2	2.449	0.2734	2.448	-0.04
3 2 0	2.550	0.2845	2.548	-0.08
3 2 1	2.646	0.2953	2.645	-0.04
4 0 0	2.828	0.3154	2.824	-0.14
4 1 0	2.915	0.3256	2.915	0.00
3 3 0	3.000	0.3348	2.998	-0.06
4 1 1	3.000	0.3348	2.998	-0.06
4 2 0	3.162	0.3529	3.160	-0.08
4 2 1	3.240	0.3617	3.239	-0.06
3 3 2	3.317	0.3700	3.313	-0.10

Table S3. Experimental and calculated diffraction data for the σ phase at 50 °C. The Miller indices correspond to the diffraction peaks labeled in **Figure 2e**.

**Miller indices (h k l)**	q_{calc} **ratio**	q_{obs} **(Å^{-1})**	q_{obs} **ratio**	$\Delta q/q_{calc}$ **(%)**
2 2 0	1.000	0.1187	1.000	0.00
2 1 1	1.038	0.1230	1.036	-0.12
3 1 0	1.118	0.1327	1.118	0.00
2 2 1	1.205	0.1429	1.204	-0.08
3 0 1	1.256	0.1490	1.255	-0.03
3 2 0	1.275	0.1515	1.276	0.11
3 1 1	1.304	0.1548	1.305	0.02
0 0 2	1.344	0.1594	1.343	-0.07
4 0 0	1.414	0.1677	1.413	-0.07
3 2 1	1.441	0.1702	1.434	-0.47
4 1 0	1.458	0.1729	1.457	-0.05
3 3 0	1.500	0.1779	1.499	-0.07
2 0 2	1.518	0.1799	1.516	-0.16
2 1 2	1.559	0.1849	1.558	-0.08
4 1 1	1.605	0.1905	1.605	0.02
3 3 1	1.644	0.1951	1.644	0.00
2 2 2	1.675	0.1987	1.674	-0.06
4 2 1	1.718	0.2036	1.716	-0.12
3 1 2	1.748	0.2075	1.748	0.01
5 1 0	1.803	0.2138	1.802	-0.07
3 2 2	1.852	0.2197	1.851	-0.06
5 0 1	1.891	0.2244	1.891	-0.01
5 1 1	1.924	0.2283	1.923	-0.03

Table S4. Diagnostic SAXS peak positions and calculated average particle radii corresponding to **Figure 2h**.

**CD at 30 °C**				
**Time (h)**	$q_{DDQC\,(00002)}$ (Å^{-1})	$\bar{R}_{DDQC}$ (Å)		
0	0.1506	25.53		
**Anneal at 40 °C**				
**Time (h)**	$q_{DDQC\,(00002)}$ (Å^{-1})	$\bar{R}_{DDQC}$ (Å)	$q_{A15\,(200)}$ (Å^{-1})	$\bar{R}_{A15}$ (Å)
1	0.1543	24.92	-	-
2	0.1561	24.64	-	-
3	0.1563	24.60	-	-
4	0.1565	24.56	-	-
6	0.1565	24.56	-	-
7	0.1563	24.60	-	-
8	0.1565	24.56	-	-
9	0.1563	24.60	-	-
11	0.1563	24.60	0.1578	24.70
13	0.1563	24.60	0.1578	24.70
15	0.1563	24.60	0.1578	24.70
20	0.1563	24.60	0.1578	24.70
168	-	-	0.1575	24.74
504	-	-	0.1578	24.70
**Anneal at 50 °C**				
**Time (h)**	$q_{DDQC\,(00002)}$ (Å^{-1})	$\bar{R}_{DDQC}$ (Å)	$q_{\sigma\,(002)}$ (Å^{-1})	$\bar{R}_{\sigma}$ (Å)
0.17	0.1556	24.72	-	-
0.5	0.1603	24.00	0.1588	24.22
1.5	0.1612	23.85	0.1593	24.15
3	-	-	0.1593	24.15
168	-	-	0.1594	24.13

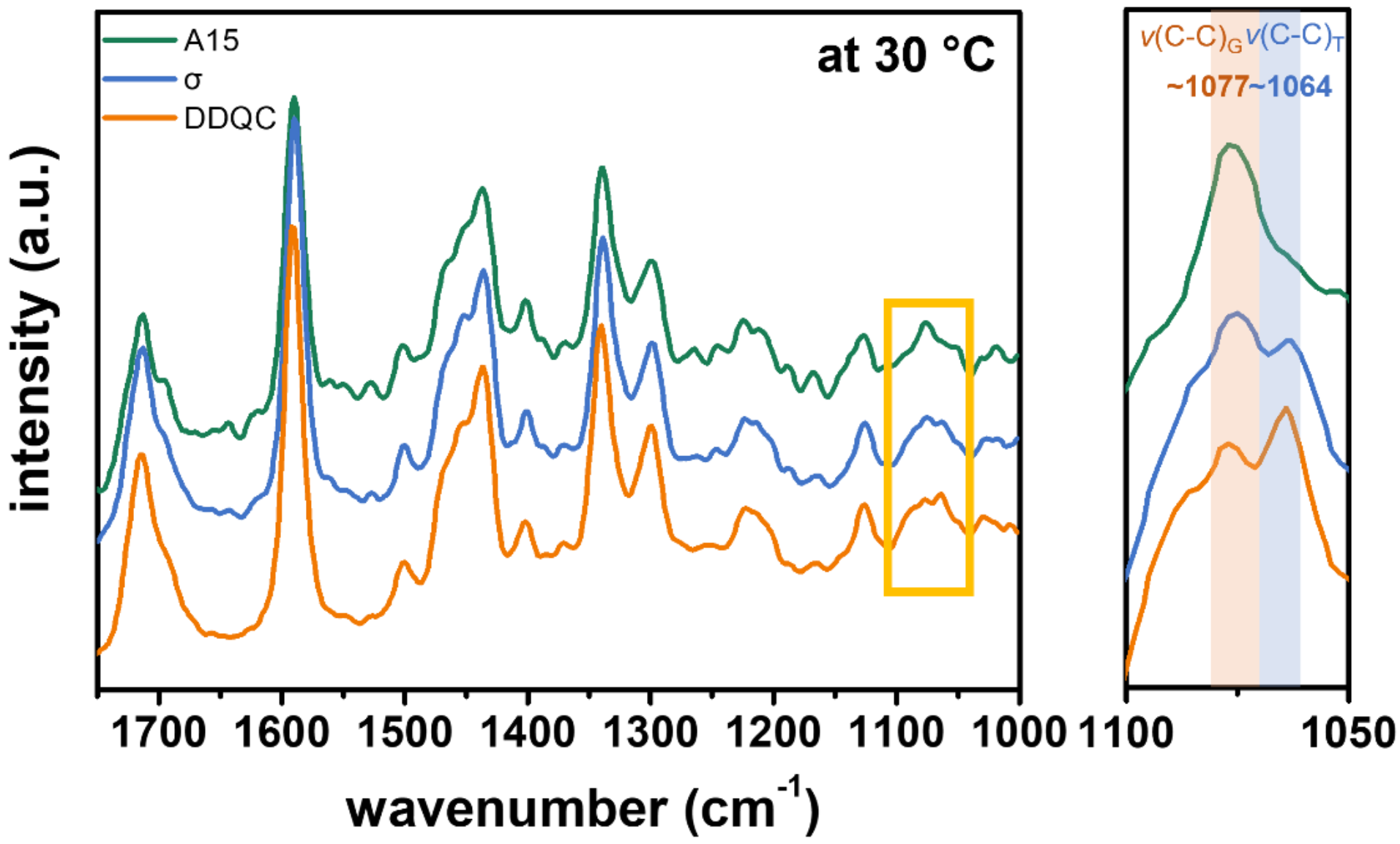

Figure S6. Raman spectra of DDQC, σ, and A15 samples measured at 30 °C. The thermal protocols were as follows: the σ sample was annealed at 65 °C for 8 h; the A15 sample was annealed at 40 °C for 504 h. The inset highlights the C-C stretching vibration region (1050–1100 cm^{-1}), showing the characteristic bands for *gauche* ($\nu(C–C)_G$, ≈ 1077 cm^{-1}) and *trans* ($\nu(C–C)_T$, ≈ 1064 cm^{-1}) conformations.[4]

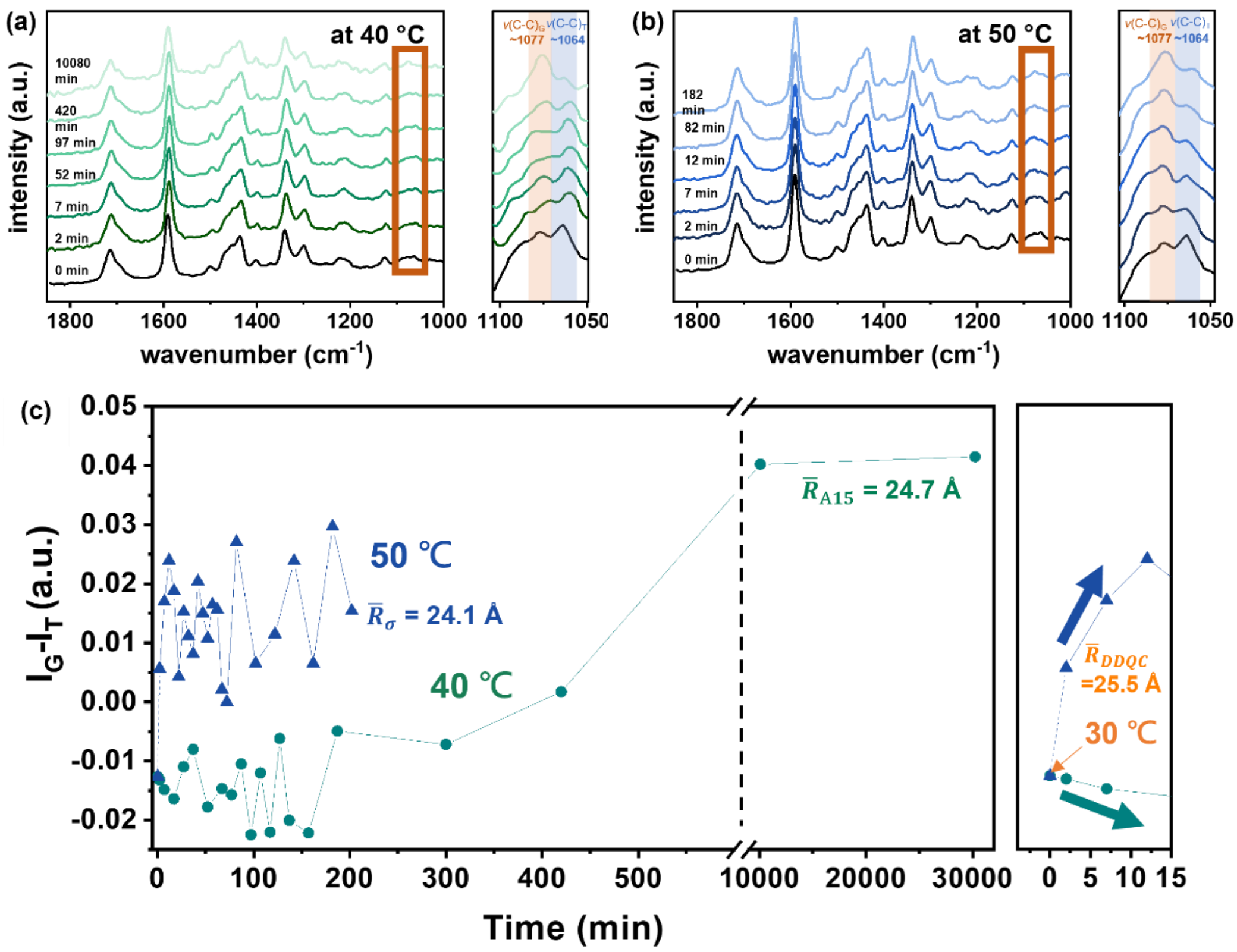

Figure S7. Time-resolved Raman analysis of chain conformational evolution during isothermal phase transformations. (a,b) Raman spectra collected during isothermal annealing at (a) 40 °C and (b) 50 °C, tracking the evolution of the C–C stretching bands associated with *gauche* and *trans* alkyl-chain conformations. (c) Time evolution of the relative Raman intensity difference, defined as $\Delta I = I[\nu(C\text{–}C)_G] - I[\nu(C\text{–}C)_T]$, at 40 °C and 50 °C. The data point labeled 30 °C represents the initial DDQC state before the temperature jump. A lower ΔI indicates an increased contribution from trans-rich, more extended chain conformations. $\bar{R}_{DDQC}$ was estimated from the DDQC (00002) reflection, whereas $\bar{R}_{A15}$ and $\bar{R}_{\sigma}$ were estimated from the A15 (200) and σ (002) reflections, respectively.

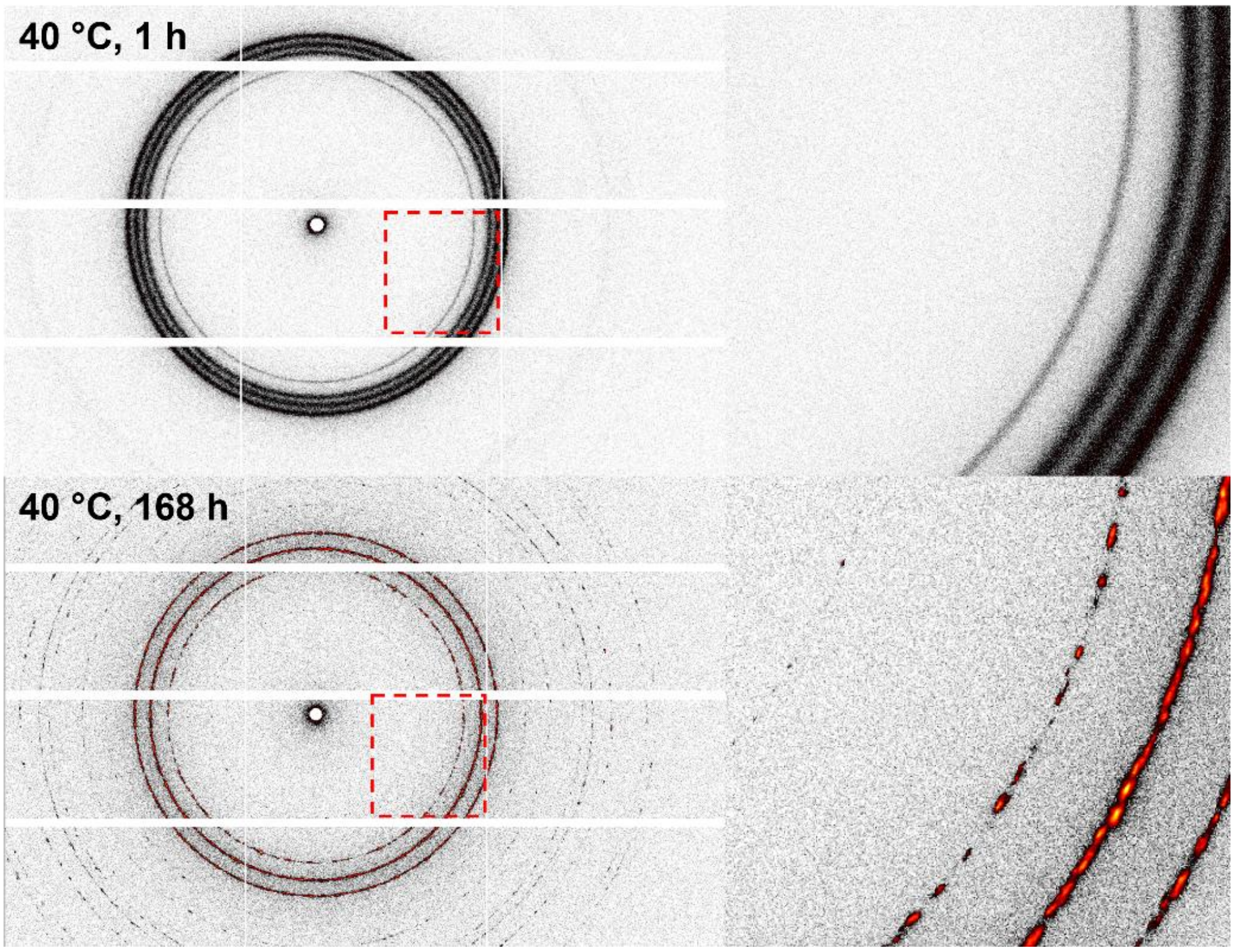

Figure S8. Evolution of 2D SAXS patterns for the sample annealed at 40 °C for 1 h and 168 h.

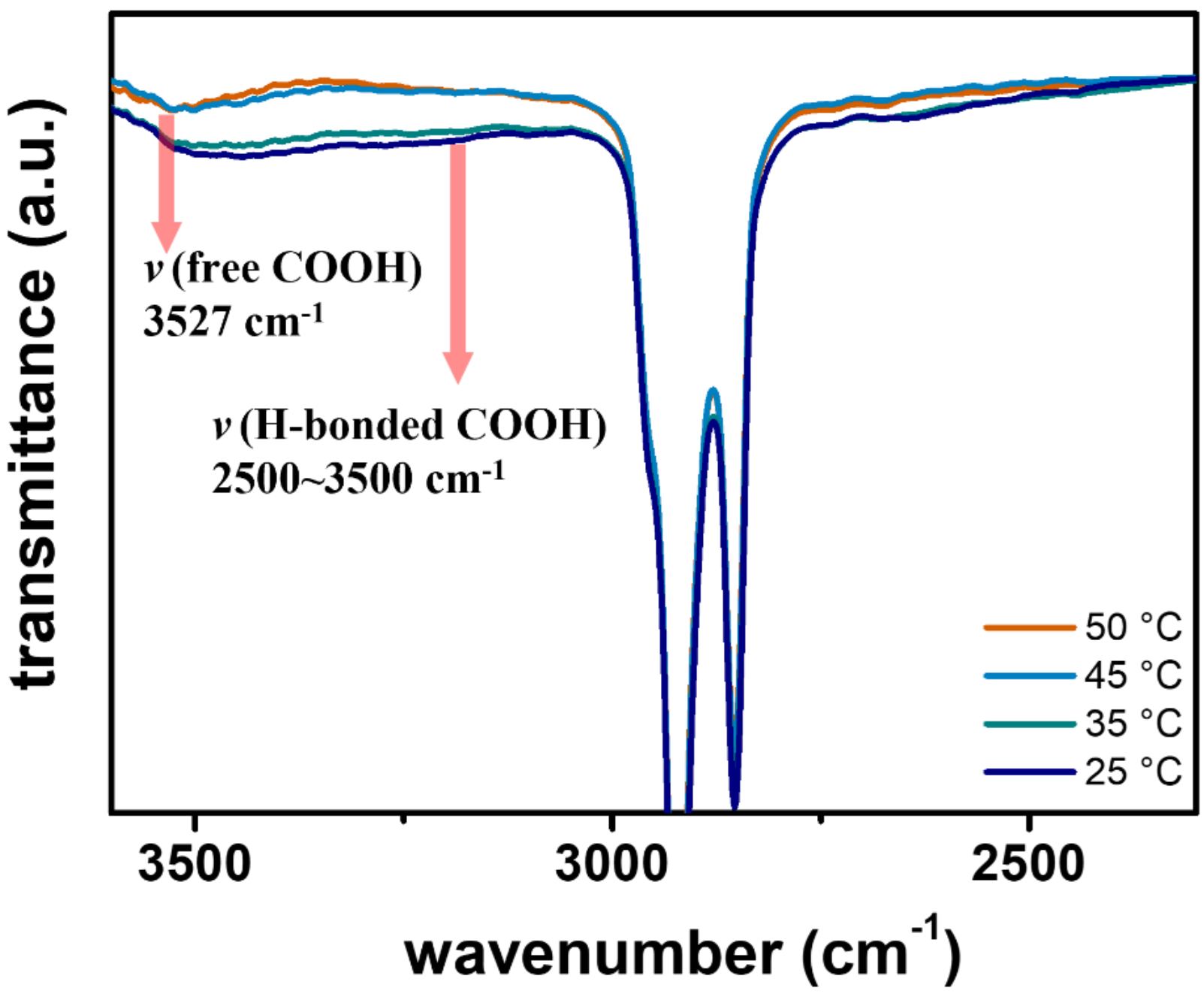

Figure S9. Temperature-dependent FTIR spectra of the **CD** assembly, recorded at 25, 35, 45, and 50 °C.

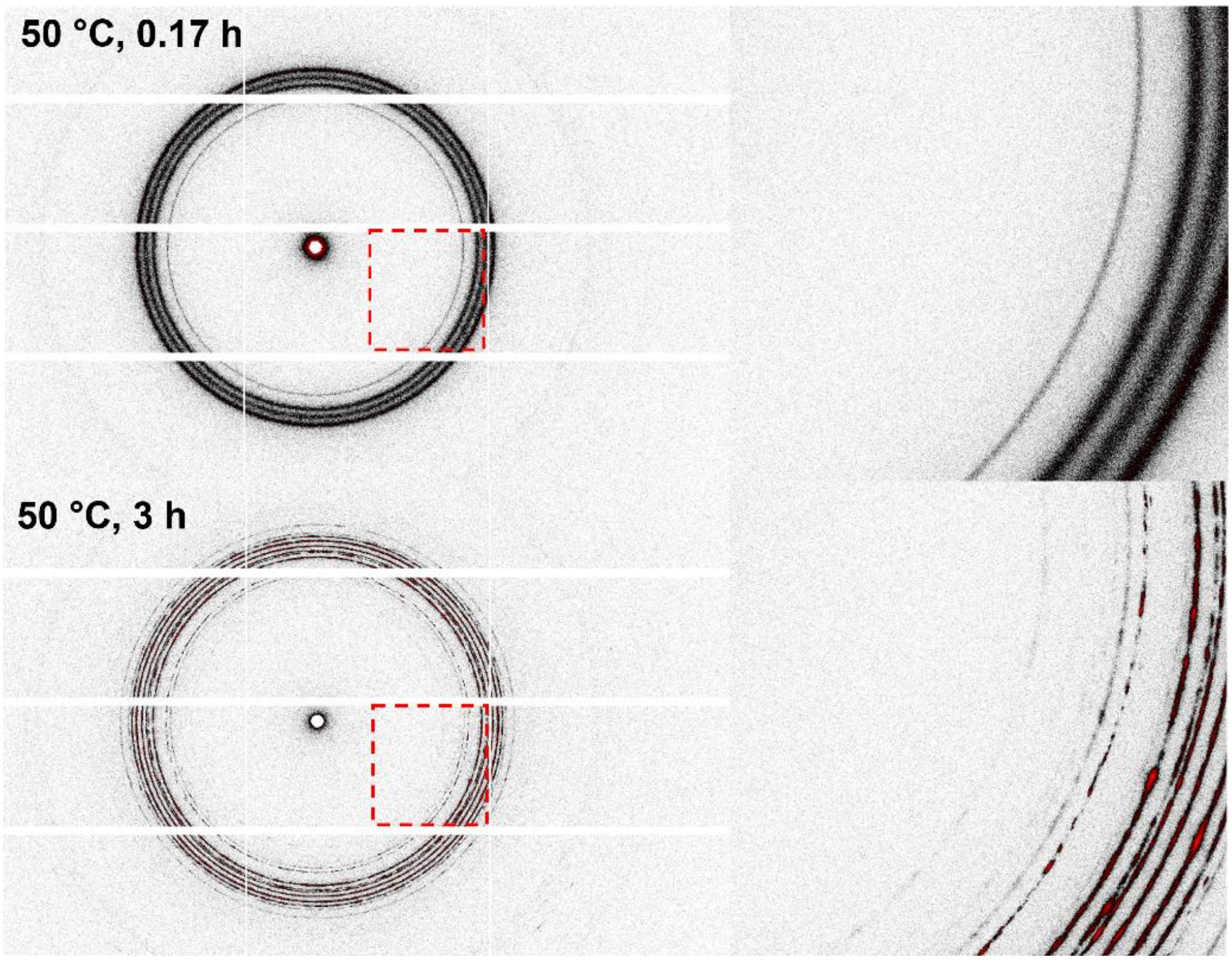

Figure S10. Evolution of 2D SAXS patterns for the sample annealed at 50 °C for 0.17 h and 3 h.

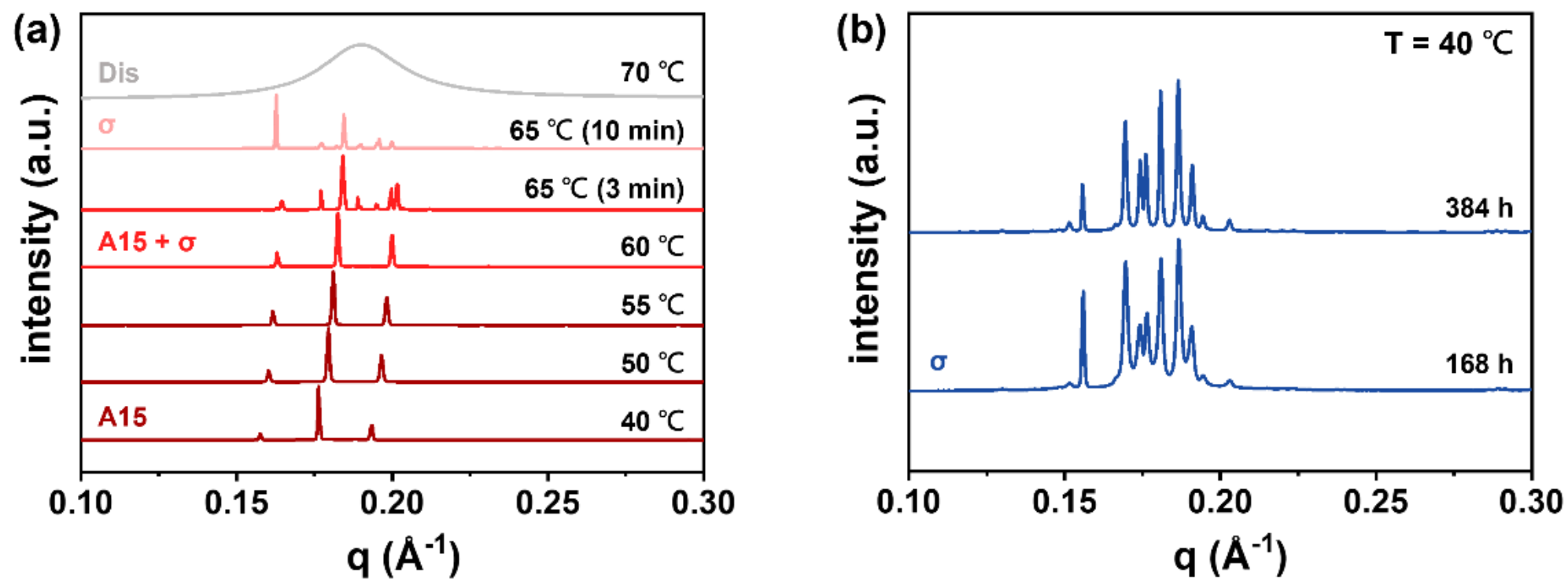

Figure S11. Investigation of the irreversibility of the A15-to-σ transition. (a) *In situ* temperature-dependent SAXS profiles of a preformed A15 sample (annealed at 40 °C for 168 h) upon subsequent heating. The sample was heated stepwise (ramp rate: 10 °C min^{-1}) with a 3 min equilibration time at each step, followed by an extended 10 min hold at 65 °C. (b) Time-resolved SAXS profiles of a preformed σ sample (annealed at 60 °C for 18 h) during subsequent aging at 40 °C for 168 h and 384 h.

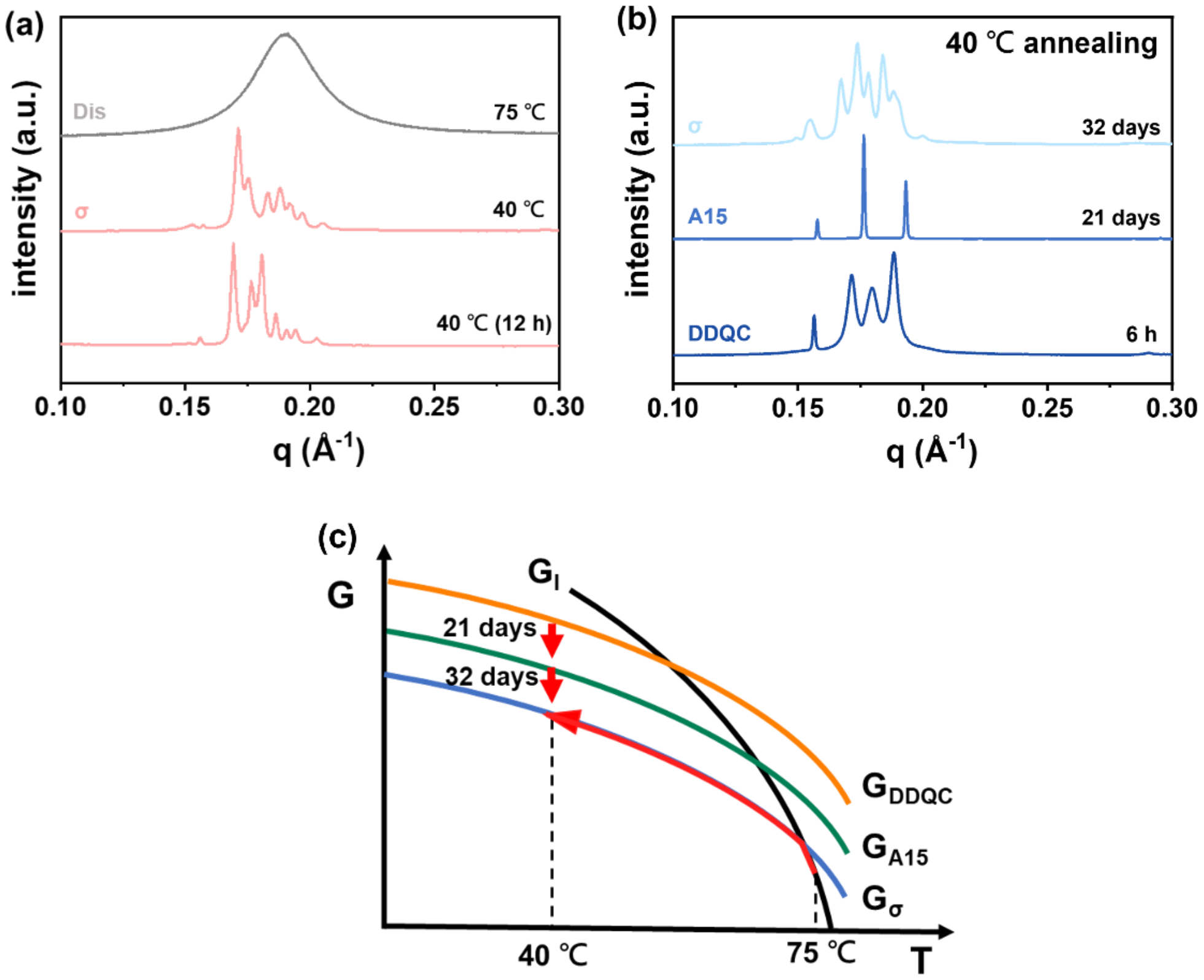

Figure S12. Direct-cooling and prolonged-annealing experiments of **CD** supporting the thermodynamic preference of the σ phase over A15 at 40 °C. (a) *In situ* SAXS profiles obtained after heating the sample to the disordered state at 75 °C, directly cooling it to 40 °C, and subsequently annealing it at 40 °C for 12 h. The σ reflections emerge upon cooling whereas no A15 reflections are observed. (b) SAXS profiles illustrating the long-time structural evolution of the DDQC-derived A15 state, which ultimately returns to the σ phase upon prolonged annealing at 40 °C. (c) Schematic illustration of the phase-transition pathways during direct cooling from the isotropic state (75 °C) to 40 °C and prolonged annealing of the DDQC-derived A15 phase. Both pathways ultimately lead to the σ phase, consistent with σ being thermodynamically favored over A15 at 40 °C.

3. Characterization of Amine-Modified CD Assemblies

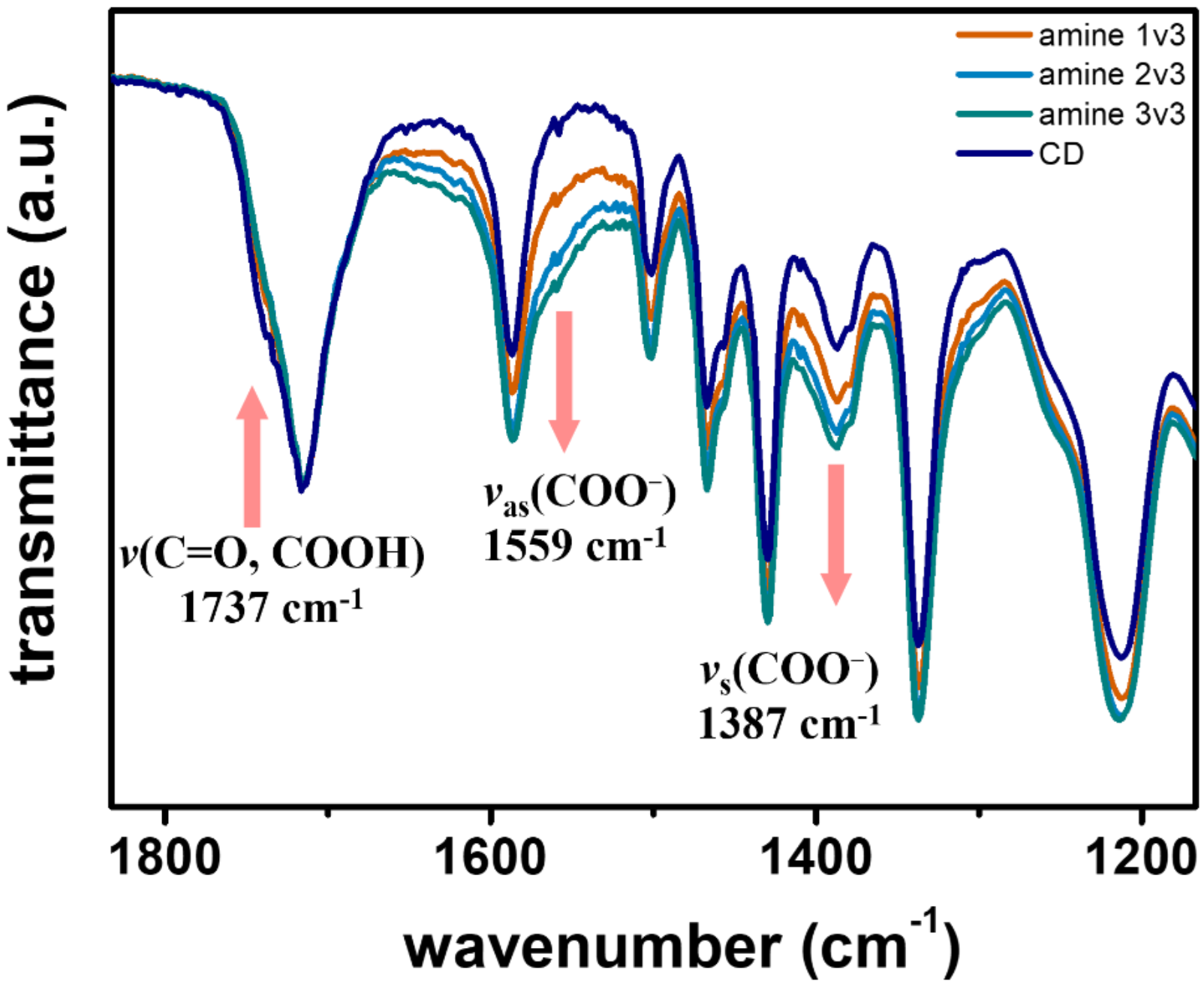

Figure S13. FTIR spectra monitoring the formation of salt bridges within the **CD** assembly upon the addition of L-alaninol (amine:**CD** molar ratios of 1:3, 2:3, and 3:3).

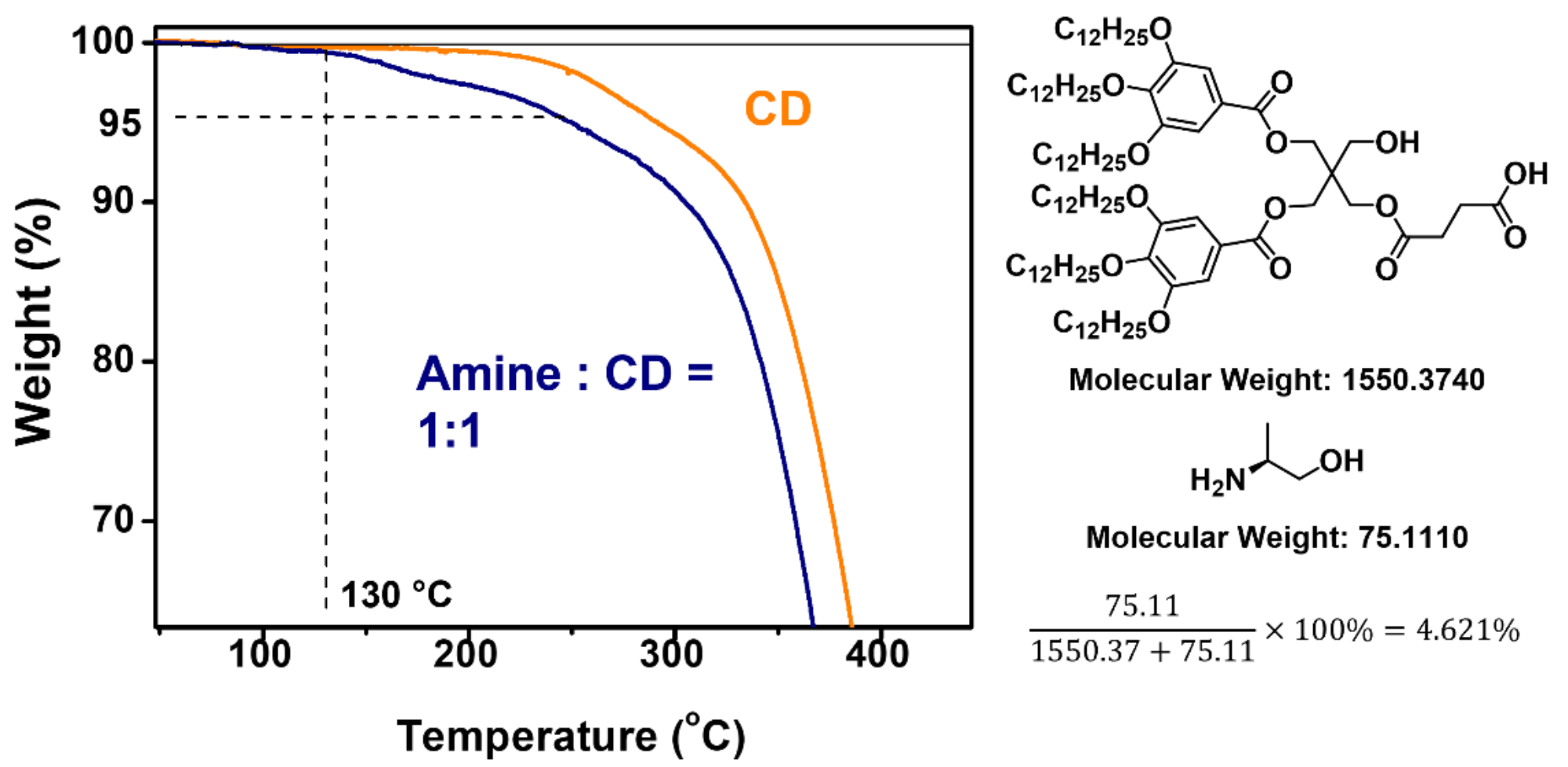

Figure S14. Thermogravimetric analysis (TGA) thermograms of the pristine **CD** (orange) and the amine-modified **CD** complex (1:1 molar ratio, blue).

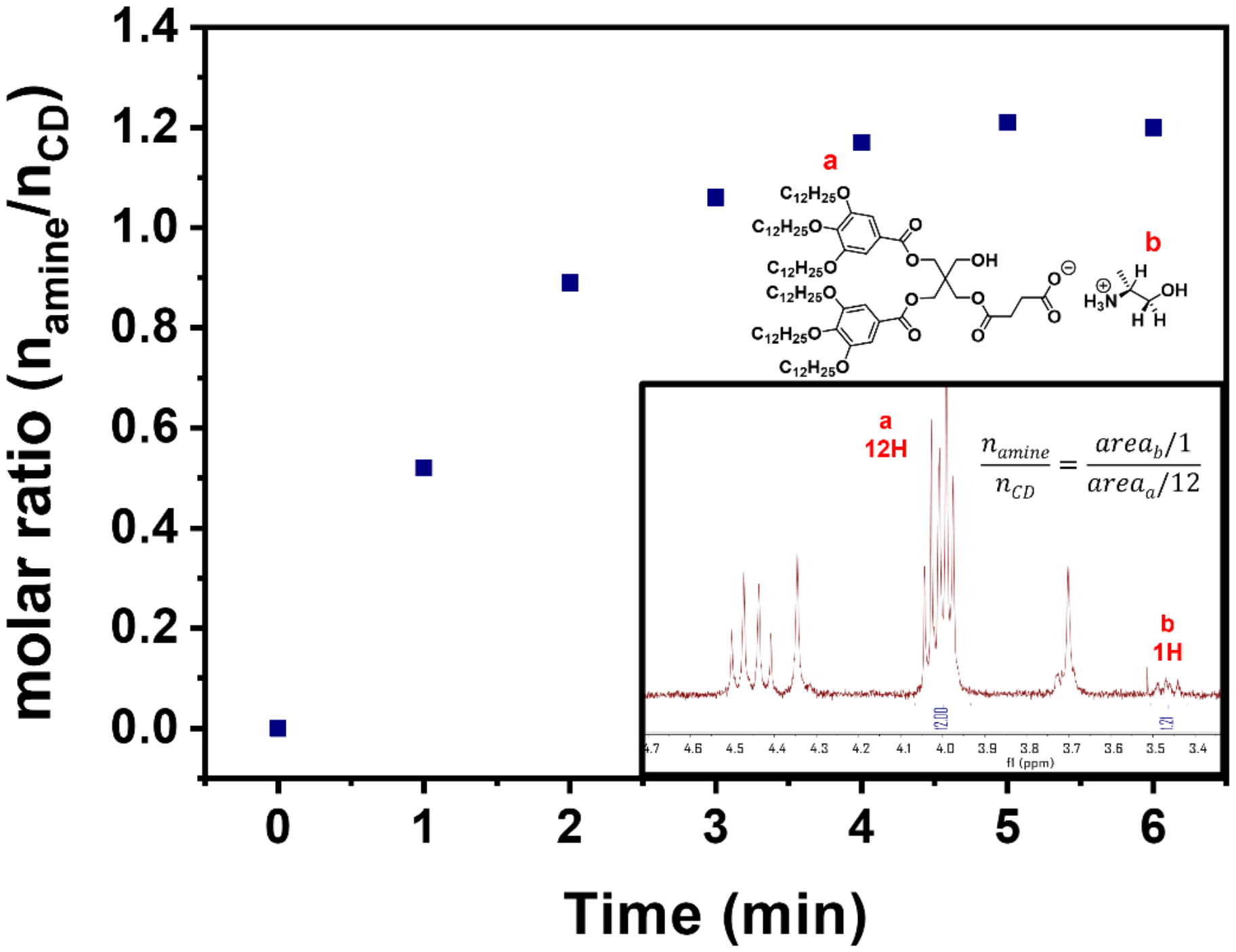

Figure S15. Time-dependent uptake profile of L-alaninol vapor. Experimental procedure: 2 mg of **CD** was spread over a 0.5 × 0.5 cm surface, heated to 90 °C, and exposed to amine vapor for different time intervals prior to dissolution in $CDCl_3$. The molar ratio (n_{amine}/n_{CD}) was determined via the integration of characteristic ^{1}H NMR signals.

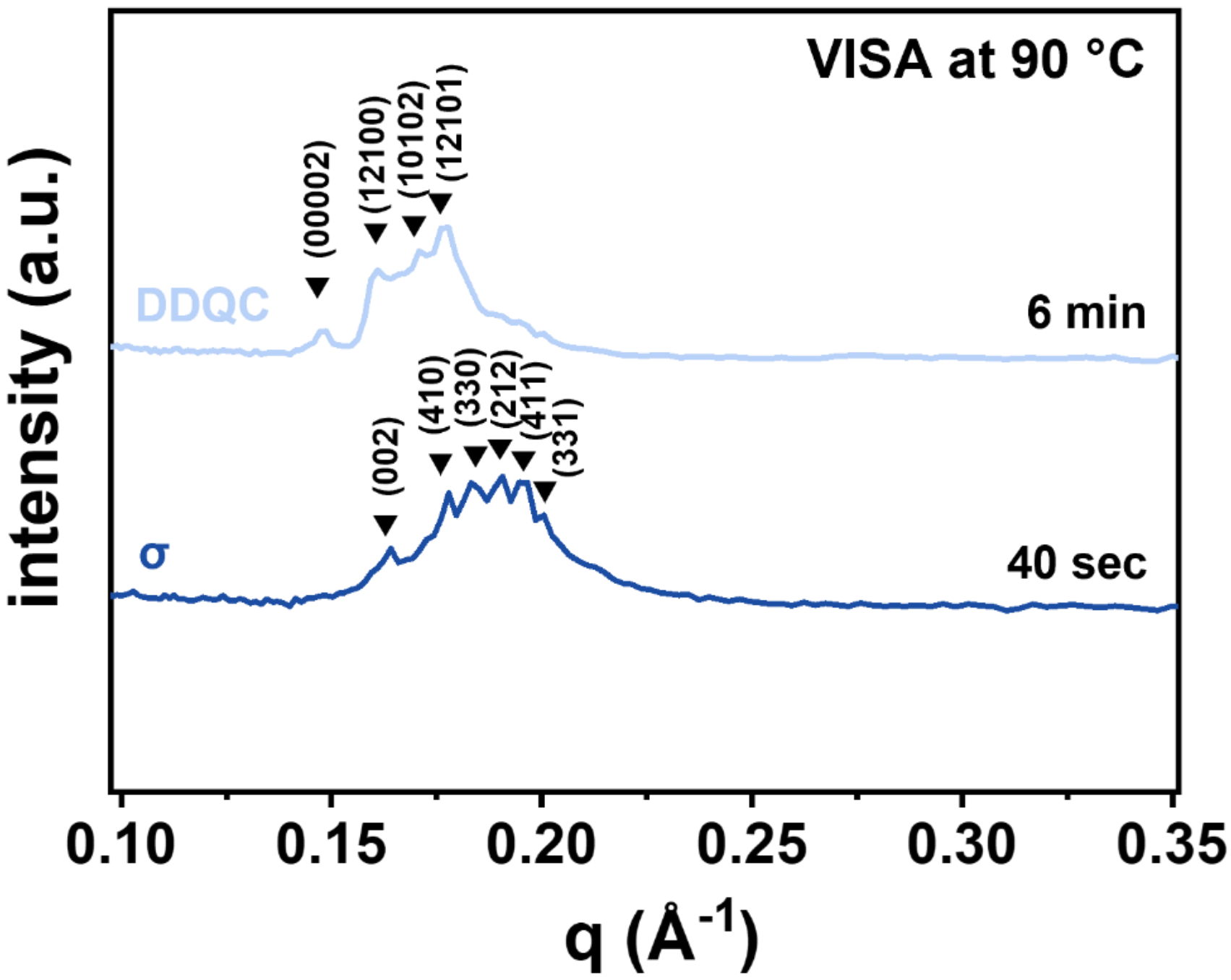

Figure S16. SAXS profiles of the DDQC and σ phases obtained at 90 °C during the VISA process. The peaks are labeled with diffraction indices corresponding to the DDQC and σ phases.

Table S5. Experimental and calculated diffraction data for the DDQC and σ phases obtained at 90 °C during the VISA process. The diffraction indices correspond to the diffraction peaks labeled in **Figure S16**.

**indices of DDQC (h_1 h_2 h_3 h_4 h_5)**	q_{calc} **ratio**	q_{obs} **(Å^{-1})**	q_{obs} **ratio**	$\Delta q/q_{calc}$ **(%)**
0 0 0 0 2	1.000	0.1472	1.000	0.00
1 2 1 0 0	1.080	0.1610	1.093	1.24
1 0 1 0 2	1.120	0.1708	1.160	3.59
1 2 1 0 1	1.190	0.1761	1.196	0.54
**Miller indices of σ (h k l)**	q_{calc} **ratio**	q_{obs} **(Å^{-1})**	q_{obs} **ratio**	$\Delta q/q_{calc}$ **(%)**
0 0 2	1.000	0.1641	1.000	0.00
4 1 0	1.085	0.1779	1.084	-0.05
3 3 0	1.116	0.1832	1.116	0.04
2 1 2	1.160	0.1907	1.162	0.18
4 1 1	1.194	0.1946	1.186	-0.69
3 3 1	1.223	0.2005	1.222	-0.07

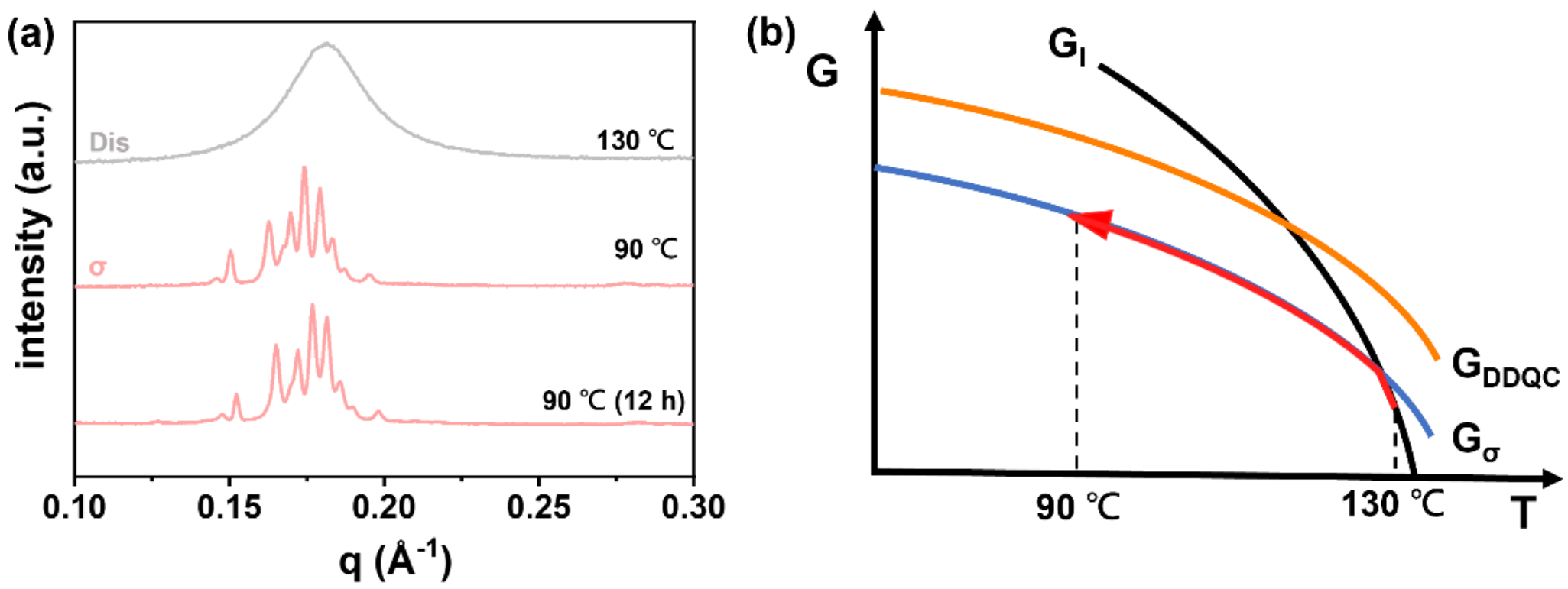

Figure S17. Direct-cooling and time-dependent SAXS analysis of the **CD**/L-alaninol assembly at saturated amine content. (a) SAXS profiles recorded after heating the **CD**/L-alaninol assembly, with amine:**CD** = 1.2:1, to the disordered state, followed by direct cooling to 90 ˚C and annealing at 90 °C. The formation and retention of the σ reflections without detectable DDQC peaks indicate that the σ phase can form directly from the isotropic melt without requiring pre-existing σ nuclei. (b) Schematic G–T diagram illustrating the phase-transition pathway during direct cooling from the isotropic melt at saturated amine content.

Table S6. Diagnostic SAXS peak positions and calculated average particle radii corresponding to **Figure 6b**.

**VISA at 90 °C**		
Time (min)	$q_{DDQC\,(00002)}$ or $q_{\sigma\,(002)}$ (Å^{-1})	$\bar{R}$ (Å)
0.66	0.1641	23.43
1	0.1594	24.13
2	0.1487	25.85
3	0.1472	26.13
4	0.1472	26.13
5	0.1472	26.13
6	0.1472	26.13

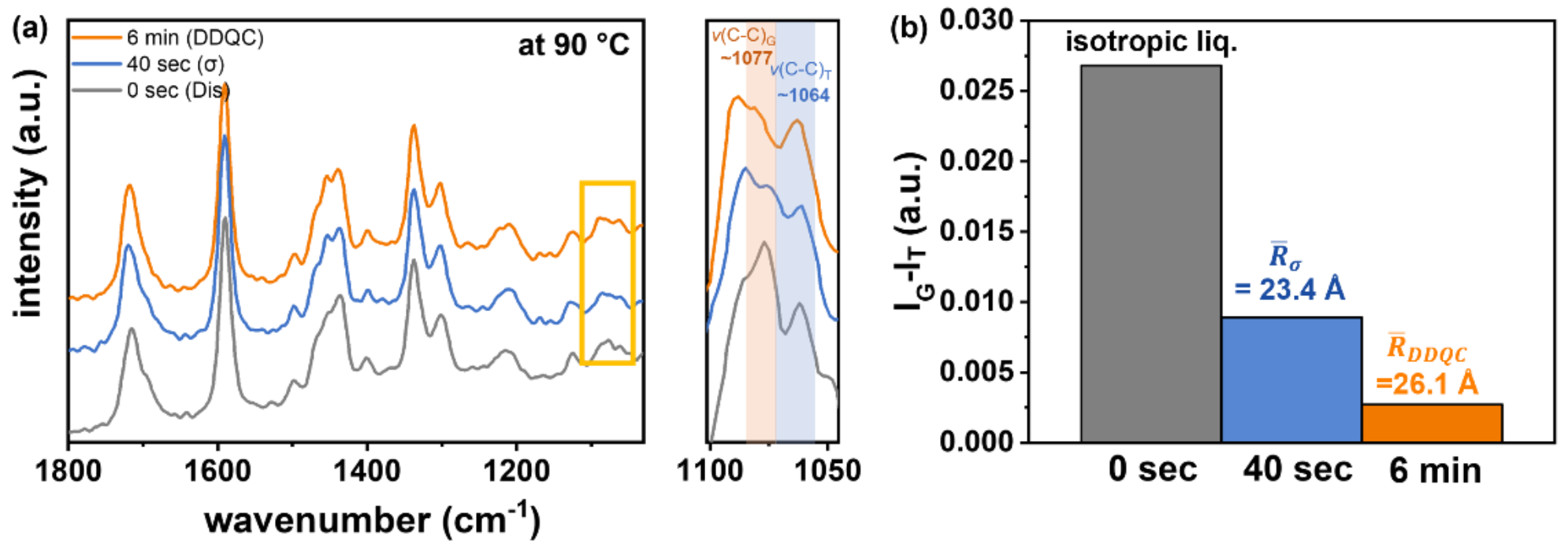

Figure S18. Raman spectra of the time-resolved transformation measured at 90 °C. (a) Raman spectra collected at different durations of the VISA treatment, corresponding to the disordered state at 0 s, the σ phase at 40 s, and the DDQC phase at 6 min. (b) The calculated relative intensity difference $\Delta I = I[\nu(C\text{–}C)_G]-I[\nu(C\text{–}C)_T]$ and SAXS-derived average particle radius, $\bar{R}$ at the indicated VISA exposure times.

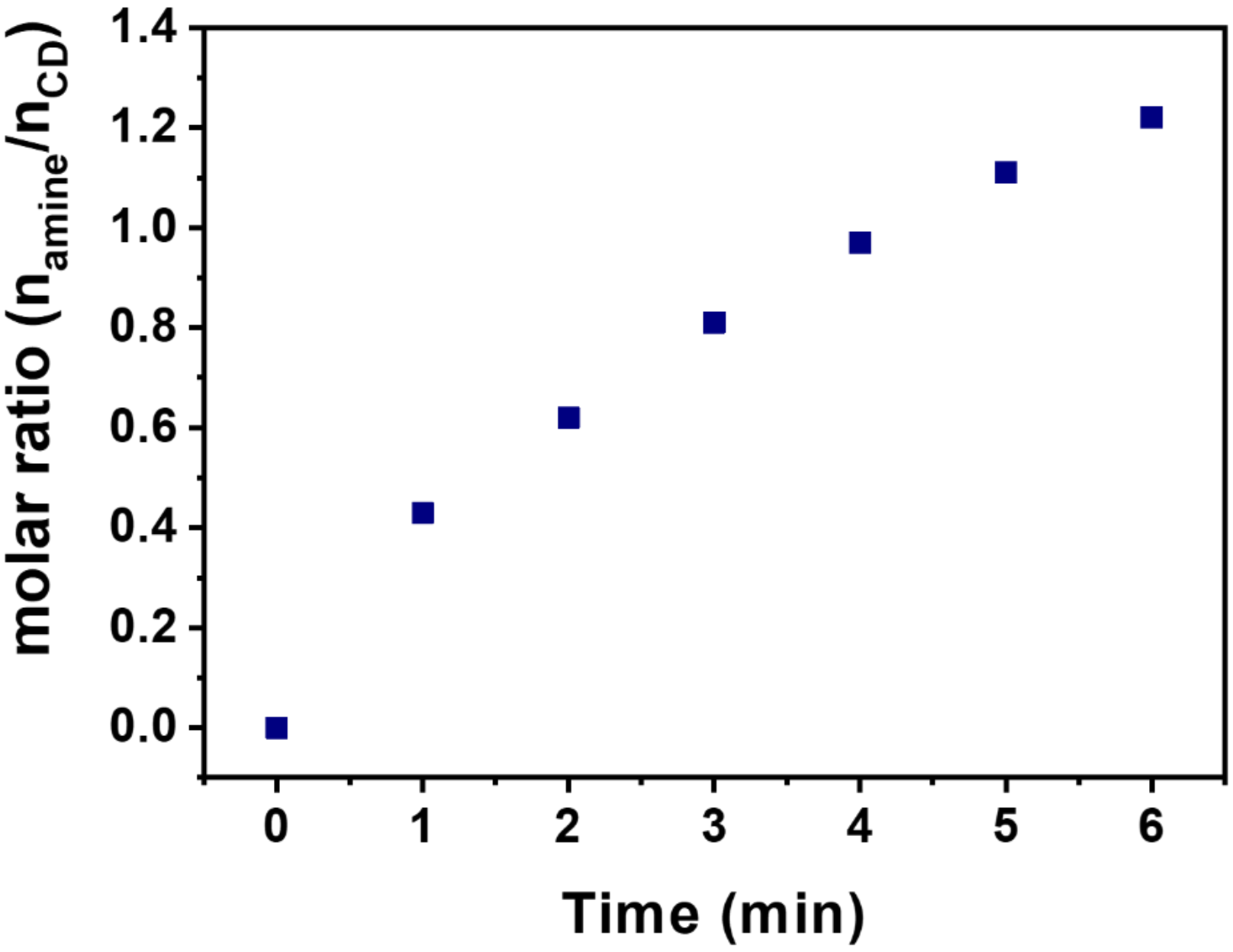

Figure S19. Time-dependent ^{1}H NMR analysis of amine uptake during VISA treatment at 115 °C.

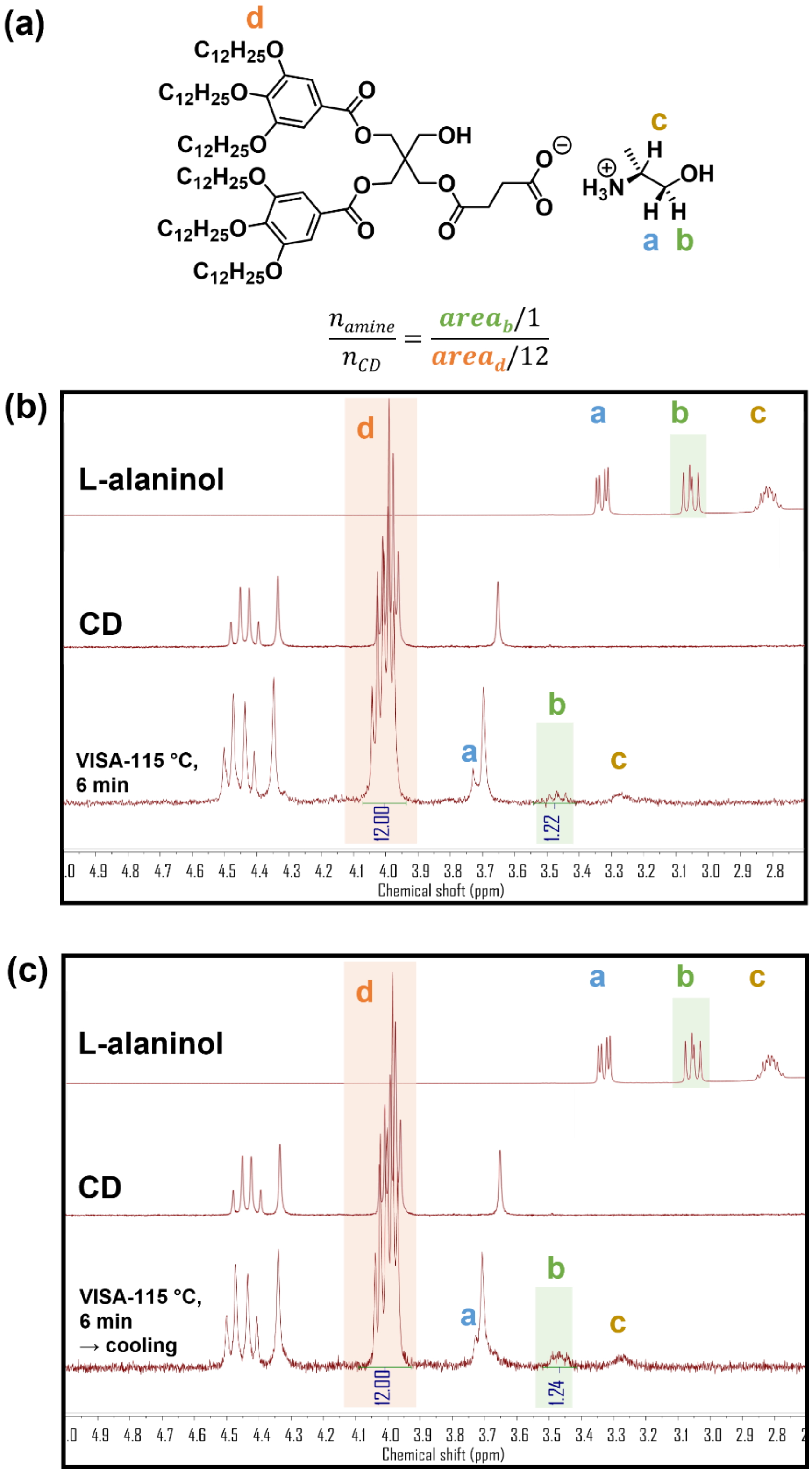

Figure S20. ^{1}H NMR analysis of amine uptake in the VISA-treated sample before and after cooling. (a) Chemical structures of **CD** and L-alaninol, with the diagnostic proton signals used to calculate the amine:**CD** molar ratio. (b,c) ^{1}H NMR spectra of the VISA-treated sample collected (b) after amine-vapor exposure at 115 °C for 6 min and (c) after subsequent cooling.

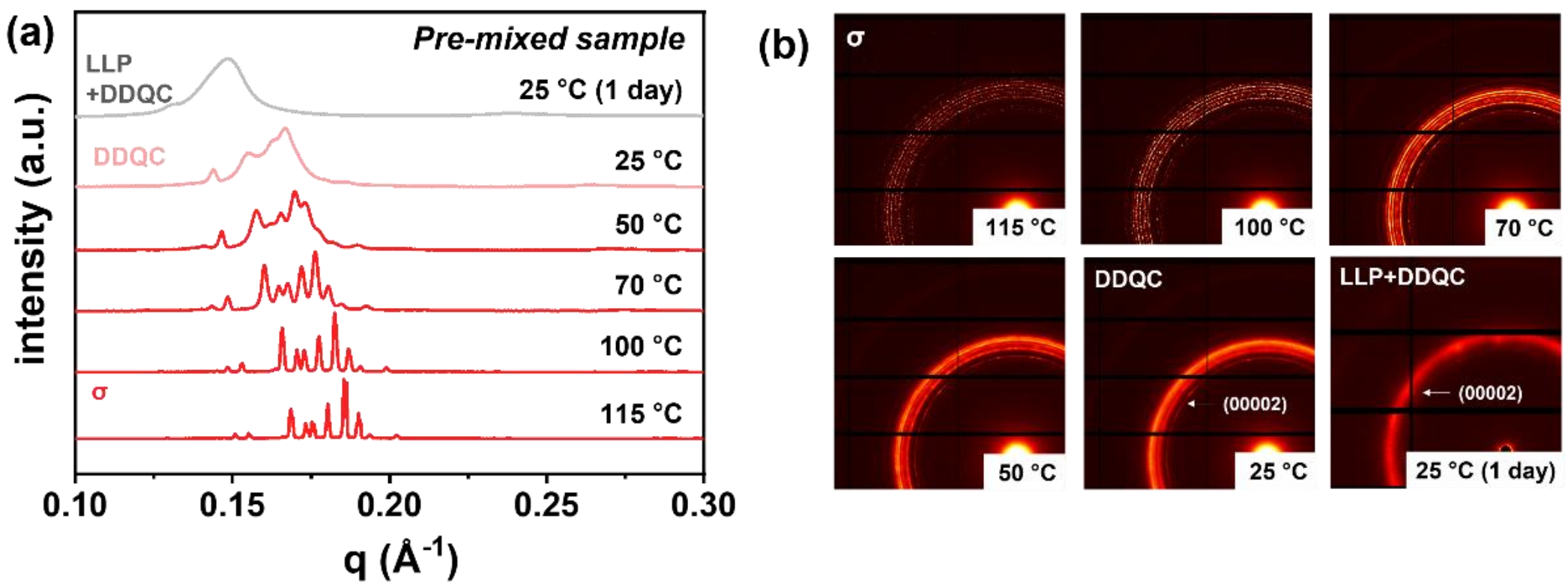

Figure S21. Cooling-induced structural evolution of a premixed **CD**/L-alaninol sample with amine:**CD** = 1.2:1. (a) Temperature-dependent SAXS profiles collected during cooling from 115 to 25 °C and after aging at 25 °C for 1 day. (b) Corresponding 2D SAXS patterns. The σ phase persists during cooling to 50 °C and transforms into the DDQC phase at 25 °C.

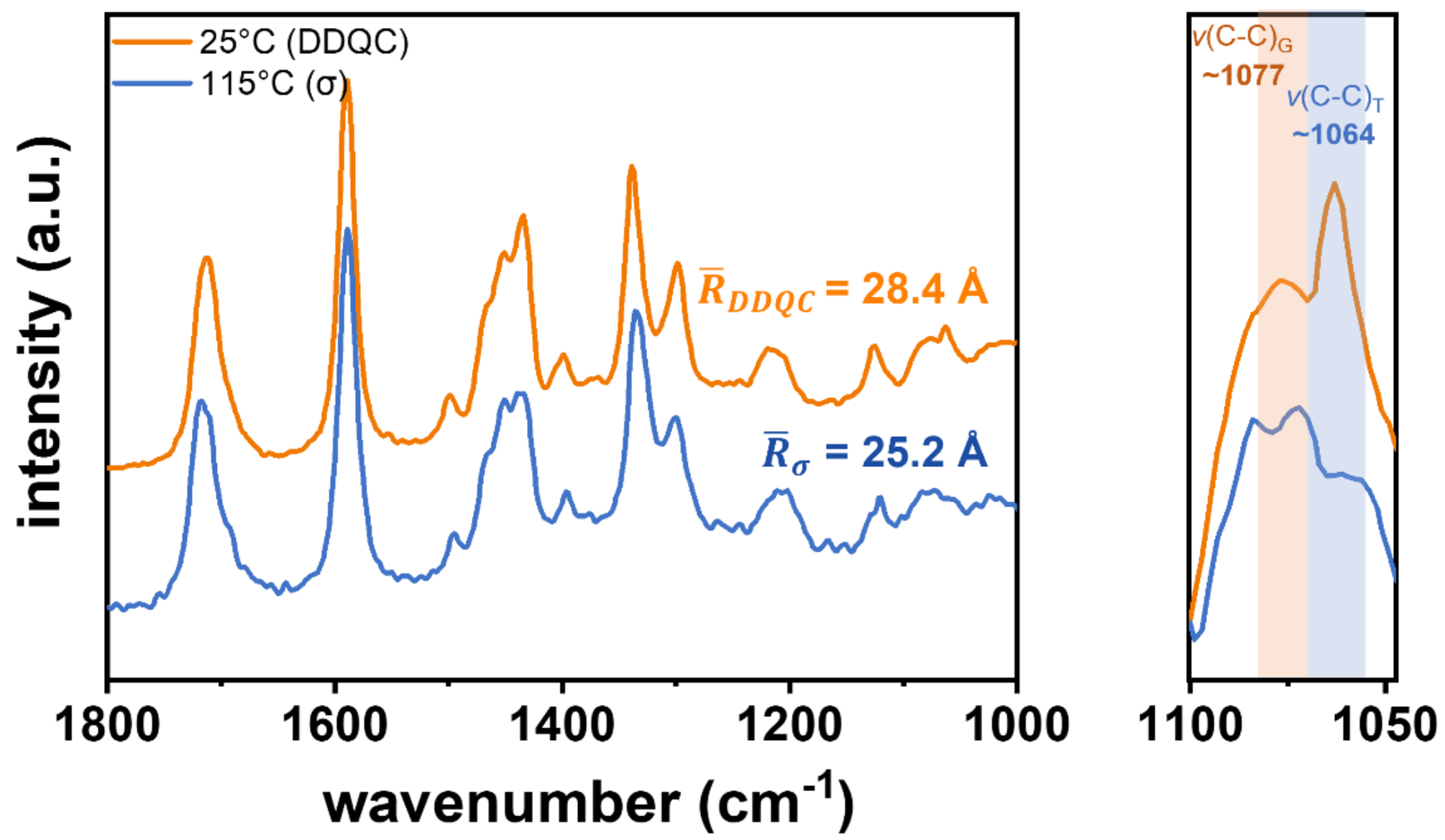

Figure S22. Raman analysis of conformational changes during cooling after VISA treatment. Raman spectra collected after VISA treatment and subsequent cooling, showing the evolution of alkyl-chain conformations during the cooling-induced σ-to-DDQC transformation. Changes in the relative intensities of the *gauche* and *trans* C–C stretching bands indicate increased *trans*-rich chain conformations, consistent with alkyl-chain extension and micellar swelling during the transition. Because the DDQC (00002) reflection is not accessible in the face-on DDQC geometry, $\bar{R}_{DDQC}$ was estimated from the DDQC (12100) reflection. $\bar{R}_{\sigma}$ was estimated from the σ (002) reflection.

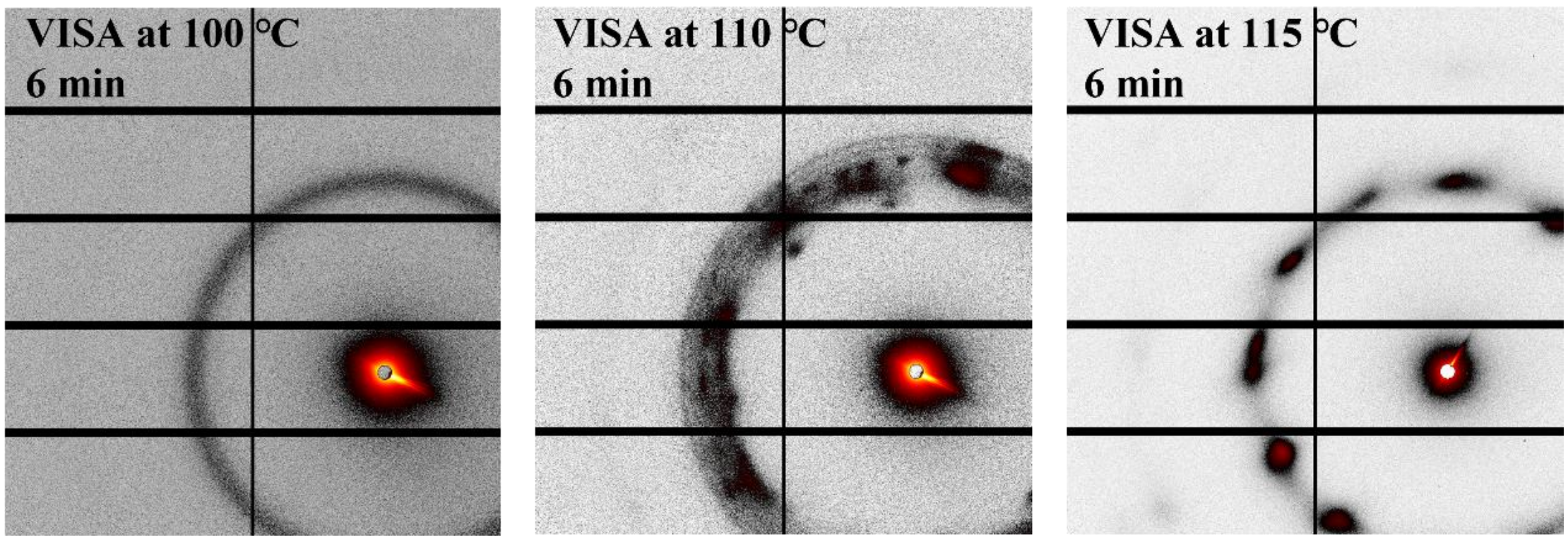

Figure S23. 2D SAXS patterns of samples subjected to the VISA process at different temperatures from the isotropic state, followed by cooling to 25 °C.

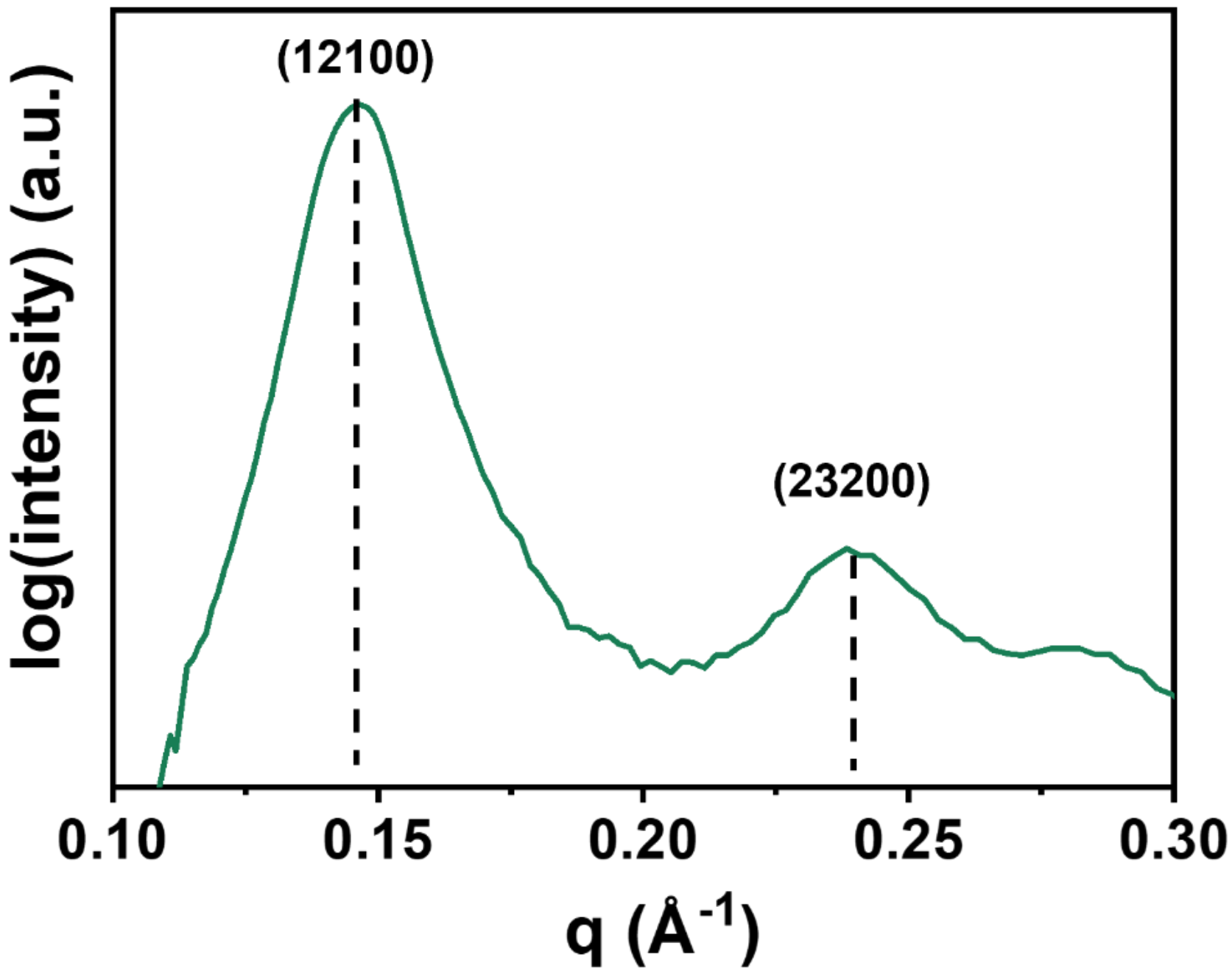

Figure S24. 1D SAXS profile of the oriented DDQC domain. The sample was prepared from the isotropic state, treated with amine vapor at 115 °C for 6 min, and measured at 25 °C.

Table S7. Experimental and calculated diffraction data for the oriented DDQC domain. The indices correspond to the diffraction peaks labeled in **Figure S24**.

**indices (h_1 h_2 h_3 h_4 h_5)**	**q_{calc} ratio**	**q_{obs} (Å^{-1})**	**q_{obs} ratio**	**$\Delta q/q_{calc}$ (%)**
0 0 0 0 2	1.00	-	-	-
**1 2 1 0 0**	**1.08**	**0.1463**	**1.080**	**0.0**
1 0 1 0 2	1.12	-	-	-
1 2 1 0 1	1.19	-	-	-
1 1 1 0 2	1.28	-	-	-
1 2 2 1 0	1.52	-	-	-
1 2 1 0 3	1.85	-	-	-
2 2 2 0 2	1.87	-	-	-
**2 3 2 0 0**	**1.87**	**0.2430**	**1.794**	**-4.1**

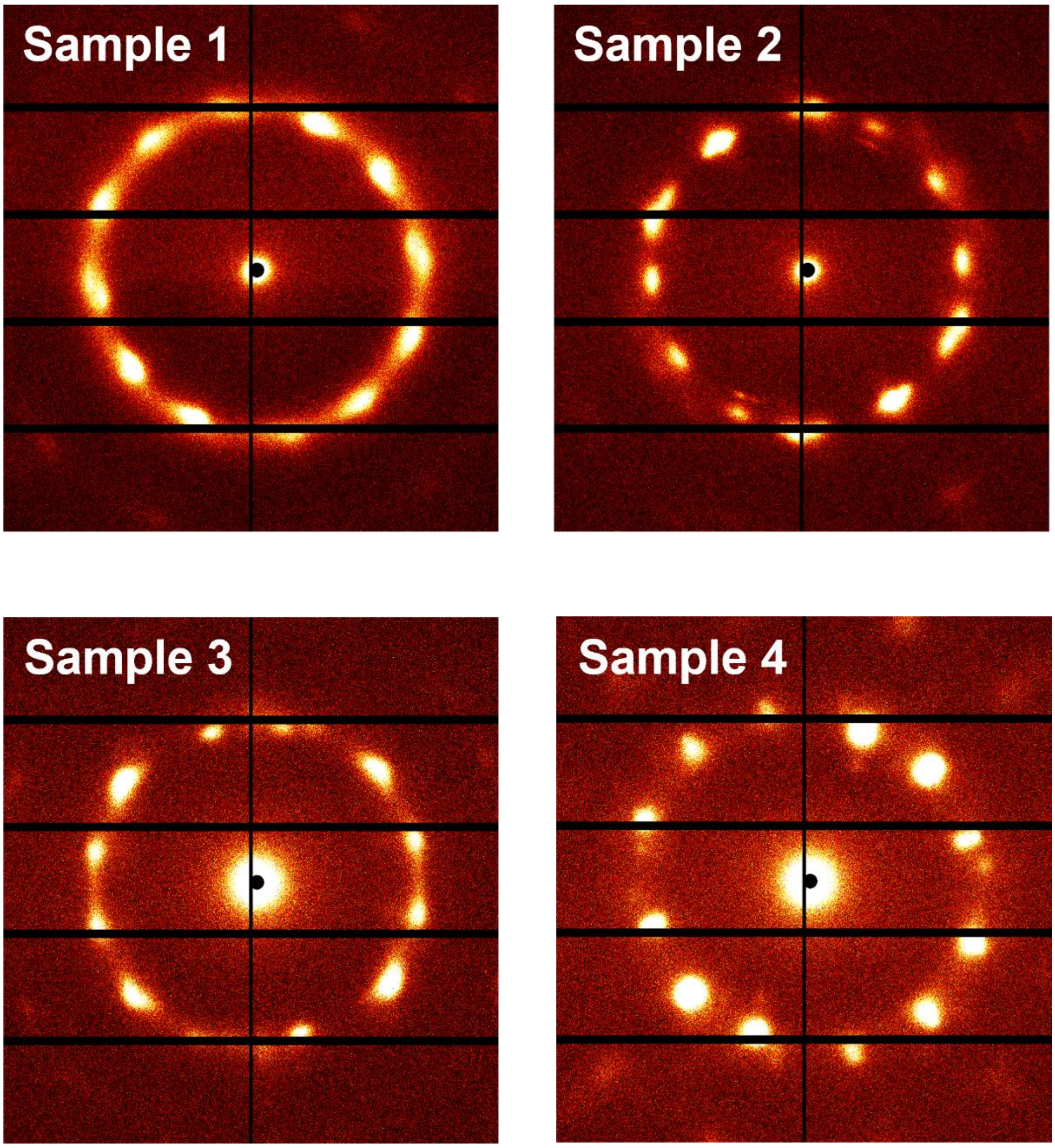

Figure S25. Reproducibility of face-on DDQC formation under the optimized VISA condition. Two-dimensional SAXS patterns obtained from four independently prepared samples after VISA treatment at 115 °C for 6 min, showing reproducible 12-fold diffraction features.

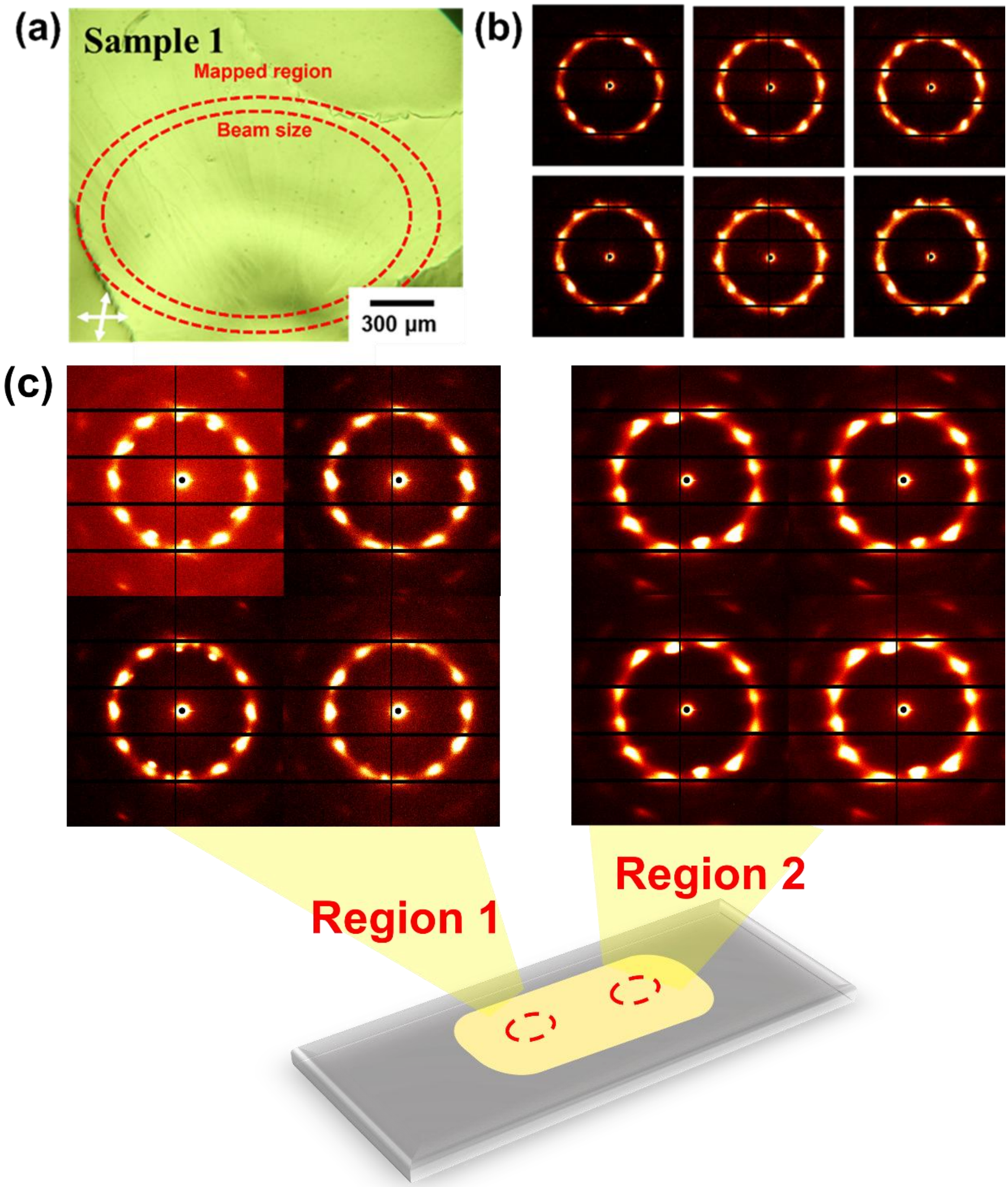

Figure S26. Spatial extent and large-area reproducibility of face-on DDQC formation. (a) Optical microscopy image of Sample 1 showing the approximate SAXS mapped region and X-ray beam footprint. (b) Two-dimensional SAXS patterns collected at six positions spanning nearly the entire crystalline domain. The well-defined 12-fold diffraction patterns exhibit nearly identical azimuthal orientations, indicating a common orientational registry throughout the mapped region. (c) Schematic illustration and corresponding 2D SAXS patterns obtained from two spatially separated regions of a larger VISA-treated sample with dimensions of approximately 10 × 5 mm.

References

1. Wang, C.-L.; Chuang, W.-T.; Lee, M.-T.; Wang, Y.-R.; Chen, S.-Y.; Huang, H.-J.; Liu, S.-Y.; Lin, J.-M.; Chen, C.-Y.; Lee, Y.-C.; Jeng, U.-S., Deactivating Symmetry Breaking of a Soft Frank-Kasper Phase via Water-Induced Conformational Ordering of a Shapeshifting Dendritic Amphiphile. *ACS Appl. Mater. Interfaces* **2025,** *17*, 31403–31410.

2. Zeng, X.; Ungar, G.; Liu, Y.; Percec, V.; Dulcey, A. E.; Hobbs, J. K., Supramolecular dendritic liquid quasicrystals. *Nature* **2004,** *428*, 157–160.

3. Lindsay, A. P.; Lewis, R. M., III; Lee, B.; Peterson, A. J.; Lodge, T. P.; Bates, F. S., A15, σ, and a Quasicrystal: Access to Complex Particle Packings via Bidisperse Diblock Copolymer Blends. *ACS Macro Lett.* **2020,** *9*, 197–203.

4. Orendorff, C. J.; Ducey, M. W., Jr.; Pemberton, J. E., Quantitative Correlation of Raman Spectral Indicators in Determining Conformational Order in Alkyl Chains. *J. Phys. Chem. A* **2002,** *106*, 6991–6998.
